# Mapping H4K20me3 onto the chromatin landscape of senescent cells indicates a function in control of cell senescence and tumor suppression through preservation of genetic and epigenetic stability

**DOI:** 10.1186/s13059-016-1017-x

**Published:** 2016-07-25

**Authors:** David M. Nelson, Farah Jaber-Hijazi, John J. Cole, Neil A. Robertson, Jeffrey S. Pawlikowski, Kevin T. Norris, Steven W. Criscione, Nikolay A. Pchelintsev, Desiree Piscitello, Nicholas Stong, Taranjit Singh Rai, Tony McBryan, Gabriel L. Otte, Colin Nixon, William Clark, Harold Riethman, Hong Wu, Gunnar Schotta, Benjamin A. Garcia, Nicola Neretti, Duncan M. Baird, Shelley L. Berger, Peter D. Adams

**Affiliations:** 1 Institute of Cancer Sciences, University of Glasgow, Glasgow, G61 1BD UK; 2 Beatson Institute for Cancer Research, Glasgow, G61 1BD UK; 3 Division of Cancer and Genetics, School of Medicine, Cardiff University, Cardiff, CF14 4XN UK; 4 Department of Molecular Biology, Cell Biology and Biochemistry, Brown University, Providence, RI 02903 USA; 5 The Wistar Institute, Philadelphia, PA 19104 USA; 6 Institute of Biomedical and Environmental Health Research, University of the West of Scotland, Paisley, PA1 2BE UK; 7 Epigenetics Program, Department of Cell and Developmental Biology, Perelman School of Medicine, University of Pennsylvania, Philadelphia, PA 19104 USA; 8 Fox Chase Cancer Center, Philadelphia, PA 19111 USA; 9 Ludwig Maximilians University and Munich Center for Integrated Protein Science (CiPSM), Biomedical Center, Planegg-Martinsried, Germany; 10 Epigenetics Program, Department of Biochemistry and Biophysics, Perelman School of Medicine, University of Pennsylvania, Philadelphia, PA 19104 USA

**Keywords:** Cell senescence, Chromatin, Tumor suppression, SUV420H2/H4K20me3

## Abstract

**Background:**

Histone modification H4K20me3 and its methyltransferase SUV420H2 have been implicated in suppression of tumorigenesis. The underlying mechanism is unclear, although H4K20me3 abundance increases during cellular senescence, a stable proliferation arrest and tumor suppressor process, triggered by diverse molecular cues, including activated oncogenes. Here, we investigate the function of H4K20me3 in senescence and tumor suppression.

**Results:**

Using immunofluorescence and ChIP-seq we determine the distribution of H4K20me3 in proliferating and senescent human cells. Altered H4K20me3 in senescence is coupled to H4K16ac and DNA methylation changes in senescence. In senescent cells, H4K20me3 is especially enriched at DNA sequences contained within specialized domains of senescence-associated heterochromatin foci (SAHF), as well as specific families of non-genic and genic repeats. Altered H4K20me3 does not correlate strongly with changes in gene expression between proliferating and senescent cells; however, in senescent cells, but not proliferating cells, H4K20me3 enrichment at gene bodies correlates inversely with gene expression, reflecting *de novo* accumulation of H4K20me3 at repressed genes in senescent cells, including at genes also repressed in proliferating cells. Although elevated SUV420H2 upregulates H4K20me3, this does not accelerate senescence of primary human cells. However, elevated SUV420H2/H4K20me3 reinforces oncogene-induced senescence-associated proliferation arrest and slows tumorigenesis in vivo.

**Conclusions:**

These results corroborate a role for chromatin in underpinning the senescence phenotype but do not support a major role for H4K20me3 in initiation of senescence. Rather, we speculate that H4K20me3 plays a role in heterochromatinization and stabilization of the epigenome and genome of pre-malignant, oncogene-expressing senescent cells, thereby suppressing epigenetic and genetic instability and contributing to long-term senescence-mediated tumor suppression.

**Electronic supplementary material:**

The online version of this article (doi:10.1186/s13059-016-1017-x) contains supplementary material, which is available to authorized users.

## Background

Cellular senescence is a stable proliferation arrest associated with an altered pro-inflammatory secretory pathway and an important tumor suppressor mechanism [1, 2]. For example, in response to acquisition of an activated oncogene, primary human cells enter a proliferation-arrested senescent state (oncogene-induced senescence (OIS)) [3–6]. Replicative senescence (RS) imposes an upper limit on the proliferative capacity of normal cells and also serves a tumor suppressor role [7, 8]. The altered secretory pathway of senescent cells, the so-called senescence associated secretory phenotype (SASP) [9–11], also contributes to tumor suppression by promoting clearance of senescent cells by the immune system [12–14].

Extensive chromatin changes are apparent in senescent cells [5, 15–24]. Importantly, the chromatin structure of senescent cells contributes to senescence-mediated tumor suppression [5, 22]. Chromatin changes in senescent cells are perhaps best illustrated by senescence-associated heterochromatin foci (SAHF) [15]. These punctate heterochromatic foci have been proposed to promote silencing of proliferation-promoting genes and/or dampen the DNA damage response in senescent cells to maintain cell viability [15, 25]. SAHF result from compaction of individual chromosomes and are enriched in a number of chromatin-associated proteins, namely histone variant macroH2a, HMGA proteins, and HP1 proteins [15, 16, 22, 26, 27]. In addition, SAHF exhibit a layered structure comprised of an H3K9me3-rich core of DNA that ordinarily replicates late in S phase in proliferating cells surrounded by an outer H3K27me3-rich domain [18].

Other studies have shown changes to the genome-wide distribution of some histone and DNA modifications in senescent cells compared with proliferating cells. In senescent cells, lamin B1 is degraded by autophagy [28–31] and this is associated with chromatin changes in and around those regions that, in proliferating cells, interact with nuclear lamins, the so-called lamin associated domains (LADs) [32]. For example, senescent cells harbor large-scale domains of H3K4me3- and H3K27me3-enriched “mesas” and H3K27me3-depleted “canyons” [20]. Mesas form at LADs, whereas canyons form mostly between LADs and are enriched in genes and enhancers. Loss of gene-repressive H3K27me3 at canyons correlates with up-regulation of key senescence genes. Some DNA methylation changes are also focused on LADs. Specifically, LADs undergo DNA hypomethylation in senescent cells [21]. Conversely, many repressed cell cycle genes gain DNA methylation flanking their promoter transcription start site and this may contribute to repression of those genes and stable senescence-associated proliferation arrest [21]. Given these precedents, other histone and chromatin modifications are also likely important in senescence.

In this regard, the abundance of a specific histone modification, H4K20me3, has been previously reported to increase in senescent cells (both OIS and RS) [33], prematurely aged (progeroid) cells [34], and physiologically aged tissues [35]. Conversely, abundance of H4K20me3 and the enzyme primarily responsible for its deposition, SUV420H2, decrease in cancer cells [36–40]. Moreover, SUV420H2 suppresses the tumorigenicity of induced pluripotent stem cells and invasiveness of breast cancer cells [40, 41]. Together, these data suggest a model whereby SUV420H2 and H4K20me3 enforce a barrier to cell transformation and tumorigenesis that is played out, at least in part, in senescent cells and aged tissues [42]. In terms of mechanism, H4K20me3 has been proposed to suppress transcription and recombination and/or control telomere elongation [41, 43–47]. A recent report showed that recruitment of H4K20me3 and the enzyme primarily responsible for its deposition, SUV420H2, to rRNA genes and IAP repeats leads to chromatin compaction at these repeats during cell quiescence and differentiation [48]. However, the genomic distribution and function of H4K20me3 in senescent cells has not been investigated. Here, we combined an epigenomic profiling approach and functional assays to better understand the role of SUV420H2 and H4K20me3 in senescence. Based on these data, we propose that elevated H4K20me3 in senescent cells contributes, at least in part, to stabilization of the senescent epigenome and genome, thereby stabilizing the senescent phenotype and, hence, long-term senescence-mediated tumor suppression.

## Results

### Senescent cells accumulate elevated levels of H4K20me3

**Fig. 1:**
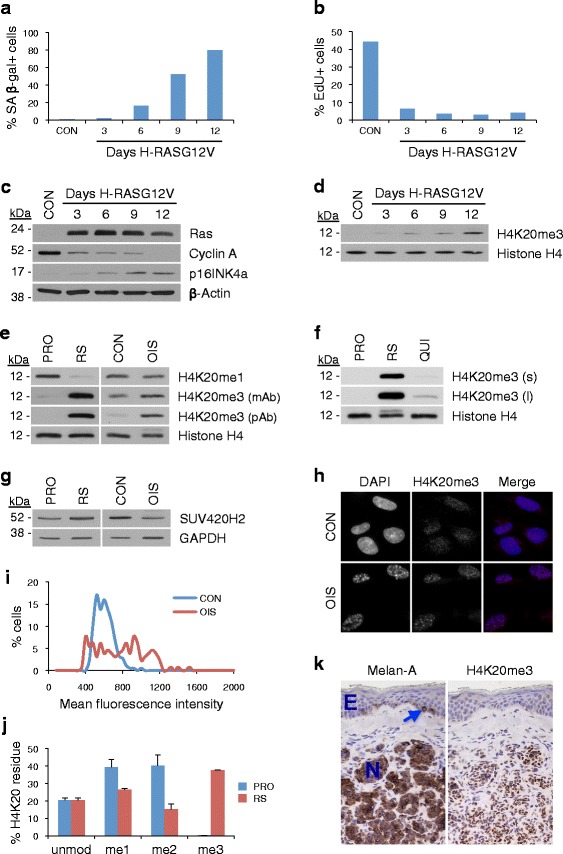
Senescent cells accumulate elevated levels of H4K20me3 in vitro and in vivo. **a** Quantification of SA β-galactosidase-positive (*SA β-gal+*) IMR90 cells 3–12 days after infection with either empty vector control (*CON*) or H-RASG12V virus. **b** Cells from **a** were pulse labeled with 5-ethynyl-2′-deoxyuridine (*EdU*) and positive cells scored. **c** Western blot of indicated proteins in whole cell extracts of cells from **a**. **d** Western blot of H4K20me3 and histone H4 in whole cell extracts from **c**, normalized for total histone H4 content. **e** Western blot of histone H4 and indicated H4K20 modifications from whole cell extracts of proliferating (*PRO*), replicative senescent (*RS*), control-infected proliferating (*CON*) and H-RASG12V-infected senescent (*OIS*) IMR90 cells, normalized for total histone H4 content. **f** Western blot of H4K20me3 and histone H4 from whole cell extracts of proliferating (*PRO*), RS and quiescent (*QUI*) IMR90 cells, normalized for total histone H4 content; *(s)* and *(l)* denote short and long autoradiographic exposures, respectively. Experiments in **a**–**f** are representative of at least five similar experiments. **g** Western blot of SUV420H2 and GAPDH from whole cell extracts of PRO, RS, CON, and OIS cells. **h** Immunofluorescent images of H4K20me3 staining in CON and OIS cells 12 days after infection. **i** Quantitative image analysis of H4K20me3 immunofluorescence in CON and OIS cells (181 CON and 129 OIS cells were scored). **j** Relative percentages of the different methylation states of H4K20 in PRO and RS cells as determined by quantitative mass spectrometry; *error bars* represent standard error of the mean. **k** Immunohistochemical images of human melanocytic nevus (*N*) and overlaying epidermis (*E*) stained with antibodies against Melan-A and H4K20me3. The *arrow* indicates a non-nevus epidermal melanocyte. Data are representative of at least ten different human nevi

In order to investigate the potential contribution of H4K20me3 to the senescence program, we first set out to better characterize the regulation and distribution of the mark in senescent cells in vitro. To accomplish this, low passage proliferating primary human IMR90 fibroblasts were infected with either control retrovirus or a virus encoding constitutively activated H-RAS (H-RASG12V) to induce OIS. As expected, compared with control-infected cells, cells expressing oncogenic H-RASG12V acquired an enlarged, flattened, senescent morphology, accompanied by an increase in senescence-associated β-galactosidase (SA β-gal) activity (Fig. 1a; Additional file 1: Figure S1a). In addition, the H-RASG12V-expressing cells underwent a proliferative arrest as evidenced by a marked reduction in 5-ethynyl-2′-deoxyuridine (EdU) incorporation (Fig. 1b; Additional file 1: Figure S1b). Consistent with a reduced proliferative capacity, the H-RASG12V-expressing cells also exhibited additional biochemical markers of cell cycle exit, including decreased cyclin A expression and p16INK4a induction, further confirming senescence (Fig. 1c). Concurrent with the onset of OIS, the mutant H-RASG12V-expressing cells exhibited a progressive increase in H4K20me3 abundance relative to total histone H4 levels (Fig. 1d; Additional file 1: Figure S1c, d). The observation that senescent cells harbor higher levels of H4K20me3 than proliferating cells was further confirmed using two additional validated antibodies in both RS and OIS cells (Fig. 1e; Additional file 1: Figure S1c, d). Consistent with previous reports [48–50], H4K20me3 levels also increased in quiescent cells relative to proliferating cells, but to only a fraction of the level observed in senescent cells (Fig. 1f). Deposition of H4K20me3 is predominantly catalyzed through the activity of the histone methyltransferase SUV420H2 [46]. Consequently, we next sought to determine whether the marked elevation of H4K20me3 levels in senescent cells occurs as a consequence of increased expression of SUV420H2. In fact, using an antibody to SUV420H2 validated against ectopic expression and knock down of SUV420H2 (Additional file 1: Figure S1e, f), only minimal changes in SUV420H2 protein expression were observed in both RS and OIS cells (Fig. 1g). Thus, the increase in H4K20me3 in senescent cells occurs independent of increased expression of SUV420H2.

To further evaluate the increase of H4K20me3 in senescence, OIS cells were subjected to indirect immunofluorescence staining for the modification. In contrast to control-infected proliferating cells, which exhibited a relatively uniform, faint, diffuse nuclear staining pattern for H4K20me3, H-RASG12V infected OIS cells displayed a more heterogeneous staining pattern, often characterized by greater overall fluorescence intensity and the presence of variably sized puncta (Fig. 1h, i). A similar increased fluorescence intensity and punctate nuclear pattern of H4K20me3 was detected in RS cells relative to low passage proliferating (PD22) cells (Additional file 1: Figure S1g).

In order to more quantitatively assess the abundance of H4K20 modifications in senescent cells, total histones were extracted from proliferating and RS cells and subjected to analysis by quantitative mass spectrometry. Whereas the trimethylated state accounted for only 0.2 % of all H4K20 residues in low passage proliferating cells, the abundance of the modification increased 190-fold to comprise 38 % of all H4K20 residues in RS cells (Fig. 1j). Of note, the increased level of H4K20 trimethylation was accompanied by a decrease in H4K20 monomethylation (H4K20me1) and dimethylation (H4K20me2), suggesting an overall conversion of H4K20me1/2 to H4K20me3 in senescent cells.

To determine whether senescent cells also harbor elevated levels of H4K20me3 under physiological conditions, the abundance of the modification was assessed in primary human tissues containing senescent cells. Human benign melanocytic nevi, neoplastic lesions of the skin comprised largely of OIS melanocytes [3, 51], were subjected to immunohistochemical evaluation of H4K20me3 abundance. Compared with the largely non-senescent keratinocytes and Melan-A-expressing melanocytes within the epidermal layer, senescent melanocytes residing within the body of the nevus displayed higher levels of H4K20me3 (Fig. 1k). This suggests that increased H4K20me3 is a bona fide epigenetic feature of cellular senescence in vivo.

### A specialized distribution of H4K20me3 in senescent cells

**Fig. 2:**
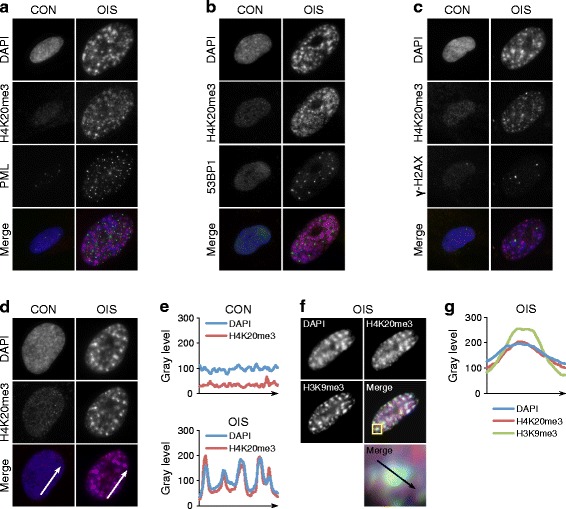
Senescence-associated heterochromatic foci (SAHF) are enriched for H4K20me3. **a** Immunofluorescent images of control (*CON*) and OIS cells co-stained with antibodies against H4K20me3 and PML. **b** Cells from **a** stained with antibodies to H4K20me3 and 53BP1. **c** Cells from **a** stained with antibodies to H4K20me3 and γ-H2AX. **d** Cells from **a** co-stained with 4′,6-diamidino-2-phenylindole (DAPI) and an antibody against H4K20me3. **e** Linescan intensity analysis of H4K20me3 and DAPI fluorescence intensity profiles along the *arrows* indicated in **d**. **f** Cells from **a** stained with antibodies to H4K20me3 and H3K9me3 and DAPI. **g** Linescan intensity analysis of H4K20me3 and H3K9me3 and DAPI fluorescence intensity profiles along the *arrow* indicated in **f**

Since H4K20me3 shows such a marked increase in senescent cells, we next wanted to know its nuclear and genomic distribution in senescent cells. First, we analyzed its distribution throughout the nucleus by immunofluorescence staining. The distribution of H4K20me3 in OIS cells showed no obvious relationship to some nuclear foci characteristic of senescent cells, namely PML nuclear bodies and DNA damage foci (γH2AX and 53BP1; Fig. 2a–c) [52–54]. However, H4K20me3 in senescent cells revealed considerable spatial overlap with SAHF (Fig. 2d) [15]. Line scan analysis of proliferating and senescent cell nuclei further confirmed the co-localization of H4K20me3 foci and SAHF in senescent cells (Fig. 2e). Moreover, in senescent cells, H4K20me3 co-localized in foci with H3K9me3, a histone modification that is highly enriched in those regions of the genome that replicate late in S phase in proliferating cells and are also folded into the core of SAHF in senescent cells (Fig. 2f, g) [18]. Similar results were obtained with RS cells (data not shown).

**Fig. 3:**
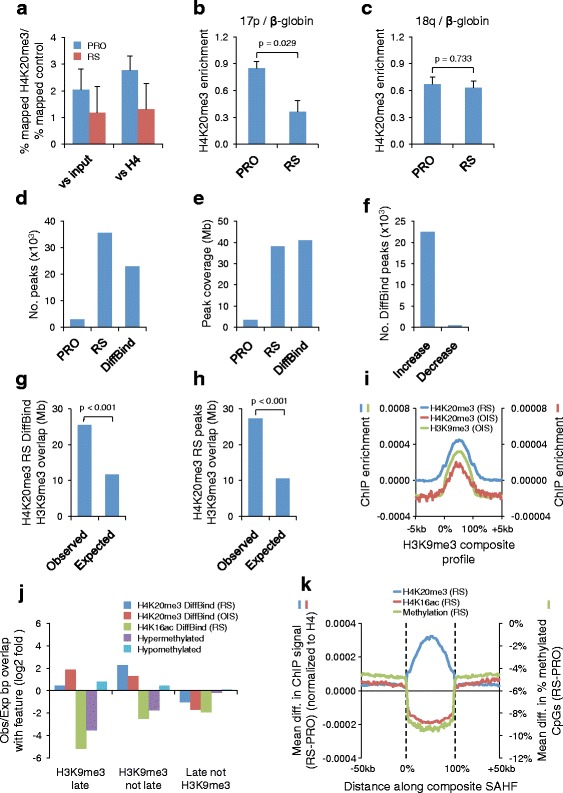
ChIP-seq confirms enrichment of H4K20me3 at SAHF in senescent cells. **a** H4K20me3 enrichment at telomeric repeat sequences relative to DNA input (*left*) and histone H4 (*right*) in proliferating (*PRO*; *blue*) and RS (*red*) cells. The *% mapped H4K20me3/% mapped control* was calculated separately for each antibody and control. Mean value (n = 2) was plotted with standard error of the mean (SEM). **b** Quantitative PCR of H4K20me3 ChIP enrichment at 17p telomeres normalized to H4K20me3 ChIP enrichment at the β-globin locus in PRO and RS cells; *error bars* represent SEM of three experiments (two experiments with the Millipore 04–079 antibody and one experiment with the Cell Signalling 5737 antibody). **c** Quantitative PCR of H4K20me3 ChIP enrichment at 18q telomeres normalized to H4K20me3 ChIP enrichment at the β-globin locus in PRO and RS cells; *error bars* represent SEM of three experiments (as in panel **b**). **d** Total number of overlapping H4K20me3 peaks identified with both antibodies (intersection) in PRO and RS cells and significantly different (false discovery rate (FDR) <0.01) peaks between PRO and RS cells determined by DiffBind. **e** Total number of base pairs comprising H4K20me3 peaks identified with both antibodies (intersection) in PRO and RS cells and significantly different (FDR <0.01) peaks between PRO and RS determined by DiffBind. **f** Number of H4K20me3 DiffBind peaks from **d** that increase and decrease in RS cells relative to PRO cells. **g** Observed overlap and expected overlap (enrichment compared to random) between base pairs covered by RS H4K20me3 DiffBind peaks with base pairs covered by H3K9me3 peaks in senescent cells (empirical *p* < 0.001). **h** Observed overlap and expected (enrichment compared with random) overlap between base pairs covered by RS H4K20me3 peaks (intersection of both antibodies) with base pairs covered by H3K9me3 in senescent cells (empirical *p* < 0.001). **i** Mean RS and OIS H4K20me3 (normalized to histone H4) and OIS H3K9me3 (normalized to input) enrichment profiles (read count) at a composite H3K9me3 peak. **j** Observed/expected overlap (log2 fold enrichment compared with random) between base pairs covered by RS and OIS H4K20me3 peaks, RS H4K16ac peaks, DNA hypermethylated in RS regions, and DNA hypomethylated in RS regions with H3K9me3-marked late-replicating regions, H3K9me3-marked not late-replicating regions, and late-replicating regions not marked by H3K9me3. **k** Mean difference (RS − PRO) in H4K20me3 enrichment, H4K16ac enrichment, and percentage of methylated CpGs at a composite H3K9me3-marked late replicating region

To define the genomic distribution of H4K20me3 at higher resolution, we applied chromatin immunoprecipitation-sequencing (ChIP-seq), using two independent antibodies highly specific for H4K20me3 (Additional file 1: Figures S1c, d and S2a, b; Additional file 2: Table S1) and an antibody to total histone H4, to proliferating and RS cells. Although the overlap of peaks obtained with the two antibodies was significant in both proliferating and RS cells, the extent of overlap was much greater in RS cells. Subsequent analyses were geared towards understanding the role of H4K20me3 in senescent cells. Since H4K20me3 has previously been reported to be enriched at constitutive heterochromatin, including telomeres [47, 49, 55–57], we first considered the possibility that increased H4K20me3 in senescent cells is largely localized to these regions. However, quantitative analysis revealed no enrichment in senescent cells compared with proliferating cells of ChIP-seq reads aligning to TTAGGG telomeric repeat sequences, regardless of whether the number of reads was normalized to input chromatin or histone H4 ChIP (to correct for any effect due to shortened telomeres in RS cells; Fig. 3a). Indeed, there was a tendency for H4K20me3 at these regions to decrease, although this was not significant. Similarly, H4K20me3 decreased, rather than increased, at subtelomeric regions (Additional file 1: Figure S3a). Moreover, ChIP-quantitative PCR (qPCR) to determine H4K20me3 enrichment in the 17p and 18q subtelomeric repeats close to the telomeric ends also showed no increase in senescent cells (Fig. 3b, c) and, in fact, a significant twofold decrease close to the chromosome 17p end (Fig. 3b). Taken together, these results indicate that the increase in H4K20me3 in senescent cells is not due to its increase at telomeric or subtelomeric sequences.

To map regions of statistically significant H4K20me3 outside of these highly repetitive sequences, domains of enrichment over background histone H4 (i.e., peaks) were identified using SICER. Only significant peaks identified with both H4K20me3 antibodies from the two independent RS experiments were considered specific and evaluated in subsequent analyses. In total, 2836 H4K20me3 peaks were identified in proliferating cells, whereas senescent cells contained 35,535 peaks (Fig. 3d). Although the mean peak length was unchanged between proliferating and senescent cells (Additional file 1: Figure S3b), the senescent H4K20me3 peaks spanned a considerably larger portion of the genome (38 Mb) than the peaks in proliferating cells (3 Mb) (Fig. 3e). An increase in the number of H4K20me3 peaks and the number of base pairs covered by H4K20me3 was also observed in OIS cells (Additional file 1: Figures S2c, d and S3c, d).

To compare the spatial distribution of H4K20me3 across the genome between proliferating and RS cells, regions of H4K20me3 differential enrichment between the intersection of the proliferating and intersection of the RS replicates were computed using DiffBind [58]. Diffbind uses edgeR to identify significantly differentially bound sites between two conditions, with multiple replicates per condition. In total, 22,955 statistically significant peaks of H4K20me3 differential enrichment were identified between the proliferating and RS cells (Fig. 3d). These peaks spanned 41 million total base pairs (Fig. 3e), accounting for approximately 1.4 % of the human genome, with a mean peak length of 1659 bp (Additional file 1: Figure S3b). Consistent with the previous intersection analysis, the vast majority of the 22,955 differentially enriched H4K20me3 peaks identified between the proliferating and RS states were more highly enriched in RS compared with proliferating cells (Fig. 3f). Similar results were obtained in OIS cells (Additional file 1: Figure S3c–e). Thus, the accumulation of H4K20me3 in senescent cells, previously observed by western blot, immunofluorescence, and mass spectrometry, is similarly observed by ChIP-seq.

In light of the previous immunofluorescence data showing co-localization of H4K20me3 and H3K9me3 in senescent cells (Fig. 2f), we first compared the genomic distribution of H4K20me3 with the genomic distribution of H3K9me3 in senescent cells, previously published by Narita and coworkers [18]. Considering either peaks of H4K20me3 determined by DiffBind or base pairs within the two antibody intersection, there was a highly significant two- to threefold enrichment of H4K20me3 overlap with H3K9me3 in RS cells and a three- to sixfold enrichment in OIS cells (Fig. 3g, h; Additional file 1: Figure S3f, g). Strikingly, the mean enrichment profiles of RS and OIS H4K20me3 at a composite H3K9me3 peak (assembled from all H3K9me3 peaks [18]) were coincident with H3K9me3 and comparable to the composite analysis of the immunofluorescence imaging data (Fig. 3i, and compare to Fig. 2g). Narita and coworkers previously reported a spatial association between late-replicating regions of the genome and H3K9me3 in SAHF, suggesting that late-replicating regions marked with H3K9me3 are repositioned during senescence to form SAHF [18]. Concordant with this, H4K20me3 was enriched at H3K9me3-marked late- and not late-replicating regions in both RS and OIS [21, 59] (Fig. 3j). However, we observed under-enrichment of H4K20me3 at those late-replicating regions not marked by H3K9me3 (Fig. 3j). To obtain a more integrated view of chromatin modifications in senescent cells, we also performed these analyses on our previously published datasets [21, 60]. A histone modification linked to chromatin decompaction, H4K16ac [61], was depleted from H3K9me3 and late-replicating regions in RS cells. Conversely, these H3K9me3-enriched and late-replicating regions tended to undergo DNA hypomethylation in RS, as indicated by under-enrichment of DNA hypermethylated regions and enrichment in hypomethylated regions (Fig. 3j). A composite analysis of all H3K9me3 regions confirmed that these regions lose DNA methylation and H4K16ac but gain H4K20me3 in both RS and OIS (Fig. 3k; Additional file 1: Figure S3h). In sum, H4K20me3 and H3K9me3 co-localize in RS and OIS cells at SAHF, whether assessed by immunofluorescence or ChIP-seq, and recruitment of H4K20me3 to these regions is specifically linked to the presence of H3K9me3, not replication timing, and coupled to coordinated changes in H4K16ac and DNA methylation.

**Fig. 4:**
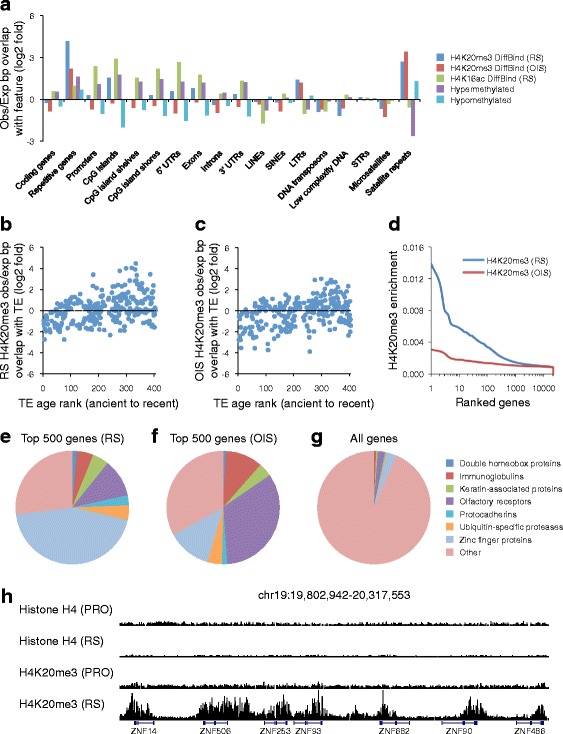
H4K20me3 is frequently enriched at ZNF and repeat class genes in senescent cells. **a** Observed overlap/expected overlap (fold log2; enrichment compared with random) between base pairs in RS and OIS H4K20me3 DiffBind peaks, RS H4K16ac DiffBind peaks, DNA hypermethylated in RS regions, and DNA hypomethylated in RS regions and base pairs covered by specified genomic features. **b** Observed overlap/expected overlap (fold log2; enrichment compared with random) of RS H4K20me3 DiffBind peaks (in base pairs) and transposable elements (TEs). The *x-axis* shows TE evolutionary order ranked from most ancient to most recent, as defined previously [62]. **c** As in **b** but using OIS H4K20me3 DiffBind peaks. **d** All coding genes ranked by RS and OIS H4K20me3 enrichment (read count) at gene body normalized to histone H4. **e** Gene families represented among the 500 gene bodies most highly enriched for H4K20me3 in RS cells. **f** As in **c** but for H4K20me3 in OIS cells. **g** Gene families represented among all genes in the genome. **h** UCSC Genome Browser view of histone H4 and H4K20me3 ChIP-seq reads aligned along a 500-kb segment of chromosome 19 in proliferating (*PRO*) and RS cells

Next, we assessed differentially enriched H4K20me3 peaks identified by DiffBind at other features of the genome sequence. H4K20me3 was selectively enriched in RS and OIS cells at some repeat elements, namely long terminal repeats (LTRs) and satellite repeats (Fig. 4a). Interestingly, although H4K20me3 was not enriched at all transposable elements (long interspersed nuclear elements (LINEs), short interspersed nuclear element (SINEs), LTRs, and DNA transposons (TEs)), it was enriched at the more evolutionarily recent TEs and under-enriched at the more ancient TEs (Fig. 4b, c) [62]. Similar results were obtained by analysis of LINEs only (data not shown). Strengthening the relationship between H4K20me3 at some classes of repeats, we also observed a marked enrichment of H4K20me3 at families of repetitive coding genes in both RS and OIS (Fig. 4a). Indeed, enrichment of H4K20me3 was most marked at a relatively small number of genes (Fig. 4d), largely comprising members of repetitive gene families, including genes encoding ubiquitin-specific proteases, protocadherins, and olfactory receptors, but most notably zinc finger proteins and olfactory receptors (Fig. 4e–h; Additional file 1: Figure S4a; Additional file 3: Datasets 1 and 2). Interestingly, this massive enrichment of H4K20me3 at repetitive genes was not markedly associated with their level of expression in RS and OIS cells determined by RNA-seq (Additional file 1: Figure S4b, c (coefficient of determination (R^2^) = 0.0108 and 0.001 for RS and OIS, respectively); Additional file 2: Table S2).

**Fig. 5:**
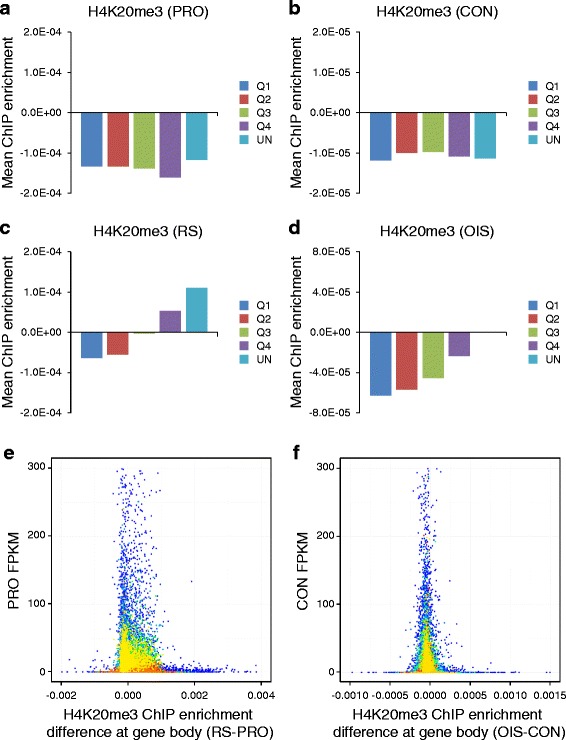
H4K20me3 genic enrichment in senescent cells occurs at lowly expressed and unexpressed genes. **a** Mean H4K20me3 enrichment (read count) normalized to histone H4 in proliferating (*PRO*) cells across the gene body (transcription start site to transcription end site) of all non-repeat class coding genes divided into quartiles on the basis of expression level (*Q1* = highest expression, *Q4* = lowest expression, *UN* = unexpressed (FPKM = 0)). **b** As in **a** but evaluating H4K20me3 enrichment in control (*CON*) cells. **c** As in **a** but evaluating H4K20me3 enrichment in RS cells. **d** As in **a** but evaluating H4K20me3 enrichment in OIS cells. **e** Scatter plot according to the R function “densCols” comparing H4K20me3 ChIP enrichment difference (*RS-PRO*) at gene bodies normalized to histone H4 and expression level (FPKM) for all genes in PRO cells (control for RS cells). *Colors* are determined by the density of data points at each point of the chart, moving from low density (*blue*) to high density (*red*). **f** As in **e** but comparing H4K20me3 ChIP enrichment difference (*OIS-CON*) and expression level (FPKM) for all genes in CON cells (control for OIS cells)

The senescence program in part reflects a pattern of altered gene expression characterized by the stable repression of proliferation-promoting genes and upregulation of SASP genes [63]. Therefore, we compared gene expression with enrichment of H4K20me3 at gene bodies (excluding the aforementioned repetitive genes) in senescent cells. In proliferating cells, there was no particular relationship between gene expression and enrichment of H4K20me3 (Fig. 5a, b); H4K20me3 was depleted from genes regardless of their level of expression. In contrast, in both RS and OIS cells, H4K20me3 was more enriched at bodies of repressed genes than expressed genes (Fig. 5c, d). However, altered H4K20me3 did not correlate strongly with changes in gene expression between proliferating and senescent cells (Pearson correlation coefficient = −0.09 and 0.09 for OIS and RS, respectively, at genes that significantly change expression between control and senescence), suggesting that the switch from proliferation to senescence is accompanied by relative enrichment of H4K20me3 mostly at repressed genes that do not change expression between proliferation and senescence. Scatter plots confirmed that the greatest increase in H4K20me3 in RS and OIS occurred at genes that were already low or unexpressed in proliferating cells (Fig. 5e, f).

### SUV420H2 reinforces OIS and promotes tumor suppression

**Fig. 6:**
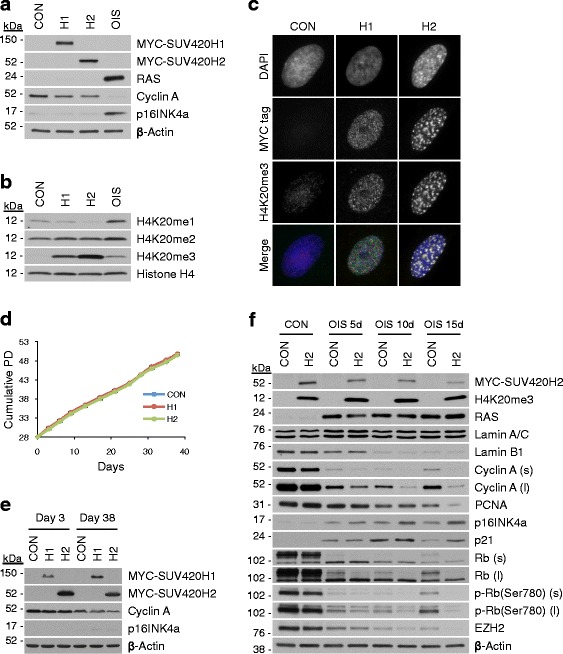
Elevated H4K20me3 levels reinforce a stable proliferative arrest in senescent cells. **a** Western blot of indicated proteins from whole cell extracts of IMR90 cells 12 days after infection with vector control (*CON*), Myc-tagged SUV420H1 (*H1*), Myc-tagged SUV420H2 (*H2*) or H-RASG12V (*OIS*). **b** Western blot of histone H4 and indicated H4K20 modifications from whole cell extracts described in **a**, normalized for total histone H4 content. **c** Immunofluorescent images of Myc-tagged SUV420H1 or SUV420H2 and H4K20me3 in CON, H1, and H2 IMR90 cells from **a**. **d** Growth curves expressed as cumulative population doublings for CON, H1, and H2 IMR90 cells measured for 38 days after infection. **e** Western blot of indicated proteins from whole cell extracts of CON, H1, and H2 IMR90 cells from **d** 3 and 38 days after infection. **f** Western blot of indicated proteins from whole cell extracts of CON and H2 IMR90 cells collected 5, 10, or 15 days after infection with either vector control (*CON*) or H-RASG12V (*OIS*); *(s)* and *(l)* denote short and long autoradiographic exposures, respectively

Since H4K20me3 increases in both RS and OIS cells, we next sought to determine whether its elevated abundance is sufficient to trigger cellular senescence. IMR90 cells were stably infected with retroviruses encoding either of the H4K20 histone methyltransferases SUV420H1 or SUV420H2 or a control virus (Fig. 6a). Ectopic expression of either SUV420H1 or H2 caused a marked elevation of nuclear H4K20me3 abundance (Fig. 6b, c). Both enzymes also produced a compensatory decrease in H4K20me1 abundance, similar to H-RASG12V (Figs. 1e, j and 6b). Of note, SUV420H2, but not SUV420H1, induced upregulation of H4K20me3 in a punctate pattern reminiscent of its localization in senescent cells and more punctate DAPI stain, suggestive of partial—but clearly incomplete—SAHF formation (Figs. 2 and 6c). Moreover, by ChIP-seq we confirmed that IMR90 cells ectopically expressing SUV420H2 exhibited a fourfold increase in H4K20me3 peaks compared with control cells (Additional file 1: Figure S5a–d). H4K20me3 peaks in SUV420H2-expressing cells showed a significant overlap with H4K20me3 peaks in RS and OIS (Additional file 1: Figure S5E) and were similarly enriched at some repetitive regions, including repetitive gene bodies, LTRs, satellites, and some more evolutionarily recent TEs (Additional file 1: Figure S5f, g). Despite these elevated levels of nuclear H4K20me3 spatially distributed similar to in RS and OIS cells, the SUV420H2- and SUV420H1-infected cells continued to proliferate normally for many population doublings (Fig. 6d), maintained cyclin A expression compared with control cells, and failed to detectably induce p16INK4a (Fig. 6a, e). We conclude that elevated H4K20me3 is not sufficient to induce acute proliferation arrest (or accelerate RS) in primary IMR90 cells.

Next, we asked whether SUV420H2 might enforce establishment and/or maintenance of OIS. Stably infected control cells or cells ectopically expressing SUV420H2 (from Fig. 6a–d) were subjected to secondary infection with either an empty vector retrovirus or virus encoding oncogenic H-RASG12V and assayed at sequential time points for markers of proliferation and senescence. Within 5 days of infection with H-RASG12V, both control and ectopic SUV420H2-expressing cells displayed features of cell cycle arrest (decreased expression of cyclin A and PCNA and reduced pRB phosphorylation (based on increased mobility in SDS-PAGE and reduced reactivity with anti-ppRB (Ser780)) and markers of senescence establishment (reduced expression of lamin B1, induction of p16INK4a and p21) (Fig. 6f). Decreased expression of EZH2, a histone methyltransferase that deposits H3K27me3 and whose downregulation contributes to upregulation of p16INK4a in senescence [64], was also observed in both control and SUV420H2-expressing cells upon H-RASG12V infection. Both control and ectopic SUV420H2-expressing cells infected with H-RASG12V displayed robust SA β-gal staining, confirming comparable induction of senescence in both cases (Additional file 1: Figure S6a). However, on examination of specific cell cycle markers 15 days after H-RASG12V infection, notable differences were observed between the control and ectopic SUV420H2-expressing cells despite equivalent expression of H-RASG12V. Compared with control cells, SUV420H2-expressing cells exhibited enhanced repression of cyclin A, PCNA, lamin B1, EZH2, and pRB hypophosphorylation and upregulation of p16INK4a and p21. Consistent with enhanced senescence in SUV420H2-expressing cells and compared with control/H-RASG12V cells, these cells consistently exhibited a lower frequency of dense crystal violet stained cell colonies appearing >15 days after infection with H-RASG12V (Additional file 1: Figure S6b). These results suggest that, although elevated H4K20me3 is not sufficient to arrest unstressed normal proliferating cells nor to accelerate RS or enhance the induction of OIS, high levels of SUV420H2 and H4K20me3 can enhance stability of the OIS program in IMR90 cells.

**Fig. 7:**
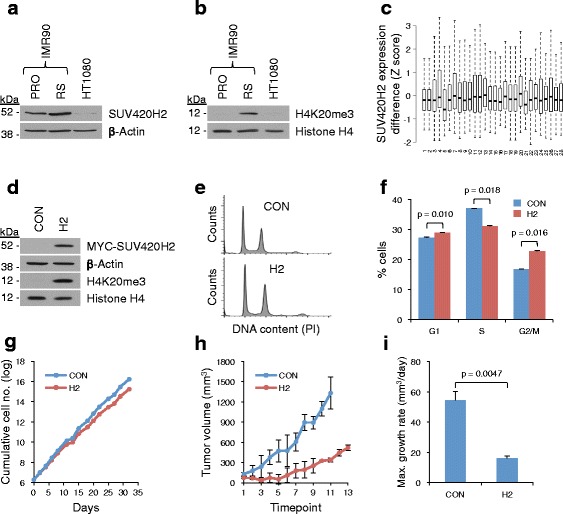
Reintroduction of SUV420H2/H4K20me3 attenuates the proliferative capacity of SUV420H2/H4K20me3-deficent HT1080 tumor cells. **a** Western blot of SUV420H2 and β-actin from whole cell extracts of proliferating (*PRO*) and RS IMR90 cells and HT1080 cells. **b** Western blot of H4K20me3 and histone H4 from whole cell extracts of PRO and RS IMR90 cells and HT1080 cells. **c** SUV420H2 expression in various human cancers relative to the reference population (either all tumors that are diploid for the gene in question or, when available, normal adjacent tissue). Data obtained from the cBioPortal for Cancer Genomics. *X-axis*, cancer type: (*1*) acute myeloid leukemia, (*2*) acute myeloid leukemia, (*3*) bladder urothelial carcinoma, (*4*) bladder urothelial carcinoma, (*5*) brain lower grade glioma, (*6*) breast invasive carcinoma, (*7*) cervical squamous cell carcinoma and endocervical adenocarcinoma, (*8*) colon and rectum adenocarcinoma, (*9*) glioblastoma multiforme, (*10*) glioblastoma, (*11*) head and neck squamous cell carcinoma, (*12*) head and neck squamous cell carcinoma, (*13*) kidney chromophobe, (*14*) kidney renal clear cell carcinoma, (*15*) kidney renal clear cell carcinoma, (*16*) kidney renal papillary cell carcinoma, (*17*) liver hepatocellular carcinoma, (*18*) lung adenocarcinoma, (*19*) lung adenocarcinoma, (*20*) lung squamous cell carcinoma, (*21*) ovarian serous cystadenocarcinoma, (*22*) pancreatic adenocarcinoma, (*23*) prostate adenocarcinoma, (*24*) sarcoma, (*25*) skin cutaneous melanoma, (*26*) stomach adenocarcinoma, (*27*) thyroid carcinoma, (*28*) uterine corpus endometrioid carcinoma. *Y-axis*, difference in SUV420H2 expression (Z score, normal/cancer). **d** Western blot of indicated proteins from whole cell extracts of HT1080 cells infected with vector control (*CON*) or MYC-tagged SUV420H2 (*H2*). **e** CON and H2 HT1080 cells were pulse labeled with 5-BrdU, fixed, and stained with propidium iodide. Fluorescence activated cell sorting (FACS) analysis to determine cell cycle distribution based on propidium iodide. **f** FACS analysis of cells from **e** to determine proportion of cells in G1, S, and G2/M phases based on 5-BrdU and propidium iodide; *error bars* represent standard deviation (SD; n = 2). **g** Growth curves expressed as log cumulative cell number for CON and H2 HT1080 cells measured for 32 days after infection. **h** Mean volumes of tumors formed after subcutaneous injection of CON or H2 HT1080 cells into CD-1 nude mice (Crl:NU-*Foxn1* ^*nu*^); n = 3 mice/group, *error bars* represent SD (representative of two independent experiments). **i** Maximum growth rates for tumors formed by CON or H2 HT1080 cells in **h** expressed as mm^3^/day; n = 3 mice/group, *error bars* represent SD (representative of two independent experiments)

To test the proliferation and hence tumor suppressive properties of SUV420H2 and H4K20me3 in another model, we turned to human HT1080 cells [65]. The cell of origin of these fibrosarcoma cells is presumably phenotypically closer to mesenchymal IMR90 fibroblasts than is the case for most commonly used epithelial-derived carcinoma cell lines. Moreover, these cells harbor an activated N-RASQ61K allele and homozygous deletion of p16INK4a [66, 67]. Consistent with a tumor suppressive role for SUV420H2 and/or H4K20me3, HT1080 cells showed decreased expression of SUV420H2 compared with proliferating and senescent IMR90 (Fig. 7a) and decreased H4K20me3 compared with RS IMR90 (Fig. 7b). In fact, mining of data in the cBioPortal for Cancer Genomics database confirmed that expression of SUV420H2 is typically downregulated in tumor compared with corresponding normal tissue (Fig. 7c). To test whether downregulation of SUV420H2 and H4K20me3 confers a direct proliferative advantage on these transformed cells, HT1080 cells were infected with either an empty vector retrovirus or virus encoding SUV420H2 and assayed for H4K20me3 abundance. As anticipated, ectopic expression of SUV420H2 induced an elevated level of H4K20me3 in the HT1080 cells (Fig. 7d; Additional file 1: Figure S6c). Ectopic expression of SUV420H2 and the commensurate increase in abundance of H4K20me3 failed to restore full senescence in the HT1080 cells (data not shown). Cell cycle analysis by propidium iodide staining and pulse labeling with 5-bromo-2′-deoxyuridine (5-BrdU) revealed a modest decrease in S phase and increase in G2/M in SUV420H2-expressing cells (Fig. 7e, f; Additional file 1: Figure S6d). The altered cell cycle distribution of the SUV420H2-expressing HT1080 cells was paralleled by a diminished rate of proliferation compared with control HT1080 cells (Fig. 7g). To test whether these effects of SUV420H2 depend on catalytic activity, we tested a SUV420H2 mutant (SUV420H2(N182A,Y217A)) previously reported to lack methyltransferase activity in mouse [68, 69]. Although this mutant was modestly under-expressed relative to the wild-type protein (Additional file 1: Figure S6e), it was profoundly impaired in its ability to elevate H4K20me3 in HT1080 cells and failed to show a trend towards decreased cells in S phase and increased cells in G2/M phase and completely failed to slow the growth of HT1080 cells (Additional file 1: Figure S6f, g), suggesting that the proliferation-inhibitory effects of SUV420H2 depend on methyltransferase activity.

To test whether elevated SUV420H2 and H4K20me3 can restrain tumor growth, HT1080 cells stably infected with either control or SUV420H2 retrovirus were injected subcutaneously into the flanks of 6-week-old CD-1 nude athymic mice (Crl:CD1-*Foxn1*^*nu*^) and tumor volumes measured at regular intervals. Mice injected with control-infected HT1080 cells formed significantly larger tumors than mice injected with HT1080 cells harboring elevated H4K20me3 through ectopic expression of SUV420H2 (Fig. 7h). Indeed, the maximum growth rate for tumors derived from control HT1080 cells was 3.8 times faster than that of the SUV420H2 expressing HT1080 cells (5.6 mm^3^/day versus 1.5 mm^3^/day; *p* = 0.0047; Fig. 7i). In sum, although SUV420H2 is unable to induce frank senescence in these p16INK4a-deficient cells, elevated levels of SUV420H2 and H4K20me3 modestly impair proliferation in culture and markedly suppress tumorigenesis in xenograft assays.

## Discussion

Several previous studies have pointed to the functional significance of H4K20me3 in senescent and progeroid cells [33, 34]. Here we have confirmed that H4K20me3 is also upregulated in senescent cells in vivo, specifically OIS melanocytes. Compared with proliferating cells, H4K20me3 is relatively enriched in both RS and OIS cells in at least three features of the genome. First, based on immunofluorescence and ChIP-seq analysis, H4K20me3 is enriched in heterochromatic SAHF. Here, H4K20me3 co-localizes with another heterochromatic modification, H3K9me3. H3K9me3 is indirectly responsible for recruitment of SUV420H2 and H4K20me3 to chromatin [49, 55], and in SAHF, H4K20me3 specifically overlapped with H3K9me3, not late-replicating DNA. Thus, H3K9me3 is likely responsible for recruitment of H4K20me3 to SAHF. Previously, we showed that telomeres are largely excluded from SAHF in RS cells [70]. In line with this initially surprising observation and enrichment of H4K20me3 in SAHF, we show here that telomeres and subtelomeres do not gain H4K20me3 in RS cells but rather show a tendency to lose this modification. Together, our results and those of Narita and coworkers [18] indicate that SAHF are assembled from late-replicating regions of the genome marked with H3K9me3 and H4K20me3, but excluding some late-replicating sequences, such as telomeres [70]. These H3K9me3-marked SAHF regions are also depleted of H4K16ac and DNA methylation. The observation that ectopic expression of SUV420H2 in cells creates H4K20me3 foci in DAPI-dense SAHF-like structures, albeit not as well formed as in RS or OIS cells, suggests that SUV420H2/H4K20me3 is partly causative for formation of SAHF. However, changes in other modifications, H4K16ac and DNA methylation, might be essential for full SAHF formation.

Second, in RS and OIS cells, H4K20me3 is markedly enriched at the gene bodies of some clusters of genic repeats, such as genes encoding ZNF proteins, olfactory receptors, and protocadherins, and also some non-genic repeats, such as LTRs, satellites, and other evolutionarily recent TEs. In fact, previous studies in proliferating cells have shown some basal enrichment of H4K20me3 at transcriptionally silent and/or repetitive sequences, including ZNF genes [44, 49, 55–57]. At such sequences, H4K20me3 and other heterochromatin marks have been proposed to suppress recombination [43–46]. Conceivably, the increase in H4K20me3 at gene repeats in senescent cells reflects this role in suppression of recombination between homologous repeat sequences. Similarly, at some non-genic repeats, elevated H4K20me3 might be involved in suppression of recombination. In addition, in light of recent reports indicating a tendency for expression and transposition of retroelements in senescent and aged cells [71–73], H4K20me3’s preferential targeting to the most evolutionarily recent and retrotransposition-competent TEs (LINEs) might reflect a role in suppression of retrotransposition, another threat to genome stability.

Third, in contrast to proliferating cells, RS and OIS cells show marked enrichment of H4K20me3 at bodies of repressed genes relative to expressed genes. Interestingly, however, this does not predominantly reflect increased H4K20me3 at genes that are expressed in proliferating cells and repressed in senescent cells. Rather, in senescent cells, H4K20me3 is gained mostly at genes that are lowly or unexpressed in both proliferating and senescent cells. This suggests that H4K20me3 does not play the role of a “switch” between proliferating and senescent cells but perhaps is more involved in a “lock down” of the already-repressed epigenome.

Consistent with this idea, while elevated SUV420H2 and H4K20me3 did not detectably impact proliferation of primary human fibroblasts, they did reinforce OIS-associated proliferation arrest. Moreover, elevated SUV420H2 and H4K20me3 markedly reduced tumorigenicity of oncogenic N-RASQ61K-expressing HT1080 cells [74]. Together, these results suggest that increased H4K20me3 does not directly induce senescence in normal human cells but can reinforce senescence and slow tumor progression in oncogene-expressing cells. While the underlying mechanisms remain to be determined, based on analysis of the ChIP-seq data, we speculate that increased H4K20me3 can stabilize both the epigenome and the genome and hence suppress epigenetic changes and genome rearrangements that promote clonal outgrowth in environments with strong selective pressure, for example, escape from OIS in vitro or clonal evolution of tumors in vivo. At least in the case of benign human nevi, OIS cells can persist in the tissue for decades [3, 51]. Long-term maintenance of tumor suppression in oncogene-expressing senescent cells likely depends on an exceptionally high level of epigenomic, transcriptomic, and genomic stability; not only because an activated oncogene is one step on the road to cancer, but also because such oncogenes often possess the ability to wreak further genetic and epigenetic havoc on the host cell. Accordingly, we hypothesize that increased H4K20me3 in senescent cells and aged tissues acts as a barrier to cancer through enhanced preservation of epigenetic and genetic stability, for example, by suppressing genome rearrangements that might allow escape from senescence and, hence, tumor progression.

Elevated H4K20me3 also caused accumulation of HT1080 cells in G2/M phase of the cell cycle and modestly slowed proliferation in HT1080 cells. The mechanism underlying this is unknown, although it might reflect the known role of histone H4 methylation in kinetochore assembly [75]. If so, elevated H4K20me3 might have multiple tumor suppressor functions.

Previous reports showed that abundance of H4K20me3 is increased in senescent cells, progeroid cells, and aged tissues [33–35] but decreased in cancer cells [36–39]. Senescent cells are known to accumulate in some aged tissues [76–78], suggesting that the increase in aged tissue might reflect accumulation of senescent cells. Alternatively, the increase in aged tissue might reflect a stress response distinct from senescence, as has been suggested for some other histone modifications [79]. On its own, elevated H4K20me3 is unable to induce senescence and proliferation arrest. This is not surprising given the concerted changes in H4K16ac and DNA methylation described here, as well as of other histone modifications [20]. However, elevated SUV420H2 and H4K20me3 can reinforce senescence-associated proliferation arrest and retard tumorigenesis of cells harboring an activated oncogene. Thus, accumulation of H4K20me3 in senescent cells and aged tissues might counter accumulation of pre-malignant oncogene-expressing cells and other damaged cells in aged tissues [80]. By extension, this can explain why H4K20me3 is decreased in cancer cells [36–40]. Downregulation of H4K20me3’s tumor suppressive function in oncogene-expressing and other damaged cells that accumulate with age is expected to confer a growth and selective advantage on the nascent cancer cell. So, while many unanswered questions remain, this study advances our understanding of the complex and contrasting regulation of H4K20me3 in senescence, aged, and cancer cells.

## Conclusions

This first comprehensive description of H4K20me3 in the chromatin landscape of senescent cells is a critical landmark contribution to understanding its previously invoked diverse functions in cell senescence, genome stability, ageing, and tumor suppression. These results corroborate the emerging view of chromatin in senescent cells as a specialized landscape that underpins the stability of the senescence phenotype. Specifically, these results implicate SUV420H2 and H4K20me3 in stable oncogene-induced senescence-associated proliferation arrest and tumor suppression.

## Methods

### Human nevus tissues

Human nevus tissues were fixed in 10 % (vol/vol) buffered formalin for 1–3 days and embedded in paraffin following routine histology procedure. Sections of unremarkable human skin and benign nevi were evaluated by a board-certified dermatopathologist (HW).

### Cell culture

IMR90 human diploid fibroblasts were obtained from the Coriell Institute (Camden, NJ, USA) and cultured in 3 % oxygen in Dulbecco’s modified Eagle’s medium (DMEM) supplemented with 20 % (vol/vol) fetal bovine serum, 2 mM L-glutamine, 25 U/ml penicillin, and 25 μg/ml streptomycin according to the suggested guidelines. IMR90 cells were considered RS when no proliferation was observed for a 2-week period following the final passage and most cells displayed senescence markers (SA β-gal, 5-ethynyl-2′-deoxyuridine (EdU) incorporation-negative, p16INK4a expression, SAHF). For induction of quiescence, cells were transferred to growth medium containing reduced serum (DMEM + 0.1 % fetal bovine serum, 2 mM L-glutamine, 25 U/ml penicillin, 25 μg/ml streptomycin). Seventy-two hours after splitting, the cells were harvested and assayed for hallmarks of proliferation arrest and senescence. The cells were considered quiescent if they no longer proliferated but also did not exhibit markers of senescence (SA β-gal activity, p16INK4a induction). HT1080 human fibrosarcoma cells were obtained from Sigma-Aldrich (St. Louis, MO, USA) and cultured in DMEM supplemented with 10 % fetal bovine serum, 2 mM L-glutamine, 25 U/ml penicillin, and 25 μg/ml streptomycin according to the suggested guidelines.

### Plasmids and retroviral infection

The following plasmids were obtained as gifts of Bill Hahn and Bob Weinberg: pBABE-puro, pBABE-puro-H-RASG12V, pBABE-neo, and pBABE-neo-H-RASG12V. pBABE-puro-Myc-SUV420H1 and pBABE-puro-Myc-SUV420H2 were generated by subcloning the respective full-length open reading frames from pC1-HA-SUV420H1 and pC1-HA-SUV420H2 (obtained as gifts of D. Alan Underhill, University of Alberta) into pBABE-puro-Myc using conventional molecular biology methods. The enzymatically inactive form of human SUV420H2, SUV420H2^N182A,Y217A^ [69], was generated by site-directed mutagenesis PCR using pBABE-puro-Myc-SUV420H2 as template. For induction of OIS, IMR90 cells were infected with control pBABE-neo or pBABE-neo-H-RASG12V and selected in 500 μg/ml neomycin. H-RASG12V-expressing cells were considered OIS 8–10 days later, at which point they expressed the same markers of senescence as RS cells (see above).

### Immunofluorescence, SAHF, and SA β-gal staining

Indirect immunofluorescence and SAHF staining assays were performed according to standard protocols and/or as previously described [15, 16, 81]. Antibodies raised against H4K20me3 (Millipore, 04–079; Active Motif, 39180), PML (Santa Cruz, sc-5621), 53BP1 (Cell Signaling Technology, 4937), γ-H2AX (Millipore, 05–636), H3K9me3 (Abcam, ab8898), and Myc (Santa Cruz, sc-40) were obtained from the respective vendors. SA β-gal staining was performed as previously described [76].

### Western blotting

Whole cell lysates were fractionated by SDS-PAGE, immobilized to PVDF, and subjected to Western blotting as previously described [81]. Antibodies raised against Ras (BD Biosciences, 610001), cyclin A (Santa Cruz, sc-751), p16INK4a (BD Biosciences, 551154), β-actin (Sigma, A1978), H4K20me3 (Millipore, 04–079 (see Additional file 1: Figure S1c for specificity); Active Motif, 39671 and 39180), histone H4 (Active Motif, 39269), H4K20me1 (Millipore, 04–735), SUV420H2 (Abcam, ab91224), GAPDH (Cell Signaling Technology, 2118), Myc (Santa Cruz, sc-40), H4K20me2 (Active Motif, 39174), lamin A/C (Cell Signaling Technology, 2032), lamin B1 (Abcam, ab16048), PCNA (Cell Signaling Technology, 2586), p21 (Abcam, ab7960), Rb (Cell Signaling Technology, 9309), phospho-Rb Ser780 (Cell Signaling Technology, 9307), and EZH2 (Cell Signaling Technology, 5246) were obtained from the respective vendors.

### Immunohistochemistry

This was performed as described previously [81]. Briefly, formalin-fixed, paraffin-embedded sections were deparaffinized, rehydrated, and blocked for endogenous peroxidases and underwent antigen retrieval according to antibody specifications. Tissues were incubated overnight with the following primary antibodies: anti-human melan A clone A103 (M7196; Dako), anti-H4K20me3 (04–079, Millipore and cs5737, Cell Signaling). Secondary antibodies used for 3,3′-diaminobenzidine (DAB)-based immunohistochemistry were either EnVision + System-HRP Labeled Polymer Anti-mouse (K4001; Dako) or EnVision + System-HRP Labeled Polymer Anti-rabbit (K4003; Dako) based on primary antibody host species. Peroxidase activity was revealed using DAB (K3468; Dako). Samples were then counterstained with hematoxylin, dehydrated, and coverslipped.

### Mass spectrometry

Quantitative mass spectrometry was performed as previously described [82]. Briefly, total histones were acid extracted from proliferating and replicative senescent IMR90 cells with H_2_SO_4_ and treated with propionyl anhydride. Bulk histones were then digested with trypsin, labeled with d10-propionic anhydride, separated by HPLC, and subjected to LC-MS/MS. The relative abundance of each histone H4 lysine 20 modification was derived using the EpiQuant analysis package [83].

### Chromatin immunoprecipitation

Proliferating and RS, control and OIS, and control and SUV420H2 IMR90 cells were cross-linked with 1 % formaldehyde, quenched with 125 mM glycine, detached by scraping, washed with phosphate-buffered saline (PBS), and collected by centrifugation. Cross-linked cells were pre-extracted with (1:1) modified nuclear lysis buffer (mNLB):immunoprecipitation dilution buffer (IPDB) (35 mM Tris–HCl pH 8.0, 75 mM NaCl, 5.5 mM EDTA pH 8.0, 3 mM EGTA pH 8.0, 0.5 % SDS, 0.5 % Triton X-100) supplemented with 10 μg/ml aprotinin, 5 μg/ml leupeptin, and 50 μg/ml PMSF and sonicated at a density of 2 × 10^7^ cells per 1 ml cold (1:1) mNLB:IPDB plus inhibitors. Sonicated chromatin solutions were cleared by centrifugation, diluted with IPDB (20 mM Tris–HCl pH 8.1, 150 mM NaCl, 2 mM EDTA, 1 % Triton X-100, 0.01 % SDS) to a final mNLB:IPDB ratio of 1:10, transferred to microcentrifuge tubes containing antibodies (from Millipore (04–079) and Cell Signaling Technology (5737); Additional file 1: Figure S1c, d) pre-bound to Dynabeads M-280 Sheep anti-Rabbit IgG magnetic beads (Life Technologies), and incubated overnight at 4 °C with rotation. The ChIP reactions were washed twice with IPDB, once with high salt wash buffer (20 mM Tris–HCl pH 8.0, 500 mM NaCl, 2 mM EDTA, 0.1 % SDS, 1 % Triton X- 100), once with LiCl wash buffer (10 mM Tris–HCl pH 8.1, 250 mM LiCl, 1 mM EDTA, 1 % NP-40, 1 % deoxycholic acid), and twice with 1× TE. Beads were aspirated to dryness, resuspended in 500 μl IP elution buffer (50 mM Tris–HCl pH 8.0, 300 mM NaCl, 10 mM EDTA pH 8.0, 1 % SDS) and 0.5 μl 100 mg/ml RNase A and incubated at 65 °C for 4–6 h. To each tube, 6 μl of 20 mg/ml proteinase K was added and the tubes were incubated at 45 °C for 12 h. ChIP DNA was purified by phenol/chloroform extraction with ethanol precipitation, resuspended with 20 μl nuclease-free dH2O, and quantified using the Qubit dsDNA HS Assay Kit and a Qubit fluorometer (Life Technologies).

### ChIP-qPCR of telomeric-adjacent sequences

The 17p qPCR assay is designed 426 nucleotides away from the beginning of the TTAGGG repeat tract. Therefore, when used to measure H4K20me3, the assay measures enrichment in both the telomere repeat tract and the telomere proximal region. The same is true of the 18q qPCR assay, which is located 444 nucleotides away from the TTAGGG repeat tract. Enrichment at each telomere was normalized to the β-globin locus (a locus that shows no substantial enrichment in either proliferating or RS cells by ChIP-seq (data not shown)), i.e., [tel]ENRICHMENT = [tel]IP/[β-globin]IP.

17p primers were: 17pA (forward), GTTTTCACCTGTTTTGGTCTTC; 17pB (reverse), GGATCCTTGCACAGGAATAAAC. 18q primers were: 18qA (reverse) TGACAGTGGTGTCCAGTGGT; 18qC (forward), CACAGGGATGGTTAGGTATCTC. Beta-globin primers were: forward, AGGACAGGTACGGCTGTCATC; reverse, TTTATGCCCAGCCCTGGCTC.

### Next-generation sequencing and analysis

ChIP-sequencing libraries were prepared using 10 ng ChIP DNA, adaptors and primers from Illumina, and the NEBNext® ChIP-Seq Sample Prep Master Mix Set 1 (New England Biolabs) according to the manufacturer’s instructions. Libraries were hybridized to Illumina flowcells using the TruSeq SR Cluster v2–cBot-GA kit and cBot instrument and subjected to 75–76 cycle, single-end sequencing using the TruSeq SBS Kit v5-GA kit and Genome Analyzer IIx sequencer (Illumina).

#### RNA-seq

Paired-end reads were aligned to the human genome (hg19) using a splicing-aware aligner (TopHat2) [84]. Only unique reads were retained (Additional file 2: Table S1). Reference splice junctions were provided by a reference transcriptome (Ensembl build 73) and novel splicing junctions determined by detecting reads that spanned exons that were not in the reference annotation. Aligned reads were processed to assemble transcript isoforms and abundance was estimated using the maximum likelihood estimate function (cuffdiff) from which differential expression and splicing are derived [85]. Genes of significantly changing expression were defined as FDR corrected *p* value ≤0.05.

#### WGBS-seq

Whole genome bisulfite sequencing data were aligned and processed to hg19 as [21]. The percentage methylation at each CpG site was calculated as [21].

#### ChIP-seq

ChIP-seq single-end reads were aligned to the human genome (hg19) using the Bowtie2 alignment software [86] (Additional file 1: Table S2). Regions of H4K20me3 and H4K16ac occupancy were determined using SICER (v1.1) [87] using a redundancy threshold of 1, window size of 200 bp, fragment size of 150, effective genome fraction of 0.75, gap size of 200, and R of 0.01. Histone H4 was used as the control for the RS and OIS models and input DNA was used as the control for the SUV420H2 ectopic expression model. For each condition (proliferating and senescent), the intersection of peaks called using both antibodies was determined using the bed tools intersect tool [88].

Differentially bound regions were determined using the R (v3.0.2) package DiffBind (v1.8.3) [58]. The count parameters used were: minOverlap = 1, bCorPlot = FALSE, insertLength = 150, score = DBA_SCORE_READS_MINUS. The analysis parameters used were: bTagwise = FALSE, bFullLibrarySize = TRUE, bCorPlot = FALSE, method = DBA_DESEQ2. Regions of significantly differential occupancy were defined as FDR corrected *p* value <0.01.

#### ChIP-seq signal

The ChIP-seq signal for any given window was calculated as the total number of fractional reads within a window divided by the window length, with the product divided by the total number of reads in the dataset divided by one million. For a normalized window the ChIP-seq signal of the control was subtracted from treatment. In Fig. 4b, g the values were normalized by the addition of the lowest signal to all signals. For all plots the pooled reads from all replicates were used.

#### H3K9me3 peaks

The senescent H3K9me3 peaks were obtained from the Gene Expression Omnibus (GEO; http://www.ncbi.nlm.nih.gov/geo/query/acc.cgi?acc=GSE38442) and converted to hg19 using UCSCs liftover tool [89].

#### H3K9me3 peak enrichment composite profiles

To generate H3K9me3 peak enrichment profiles, the area between each H3K9me3 peak was divided into 50 windows of equal size (each corresponding to 2 % of the total peak region). Fifty additional 100-bp windows were prepended (appended) to the start (end) of the peak region to provide genomic context. The average normalized ChIP-seq signal was then calculated for each window. H4K20me3 and H3K9me3 were normalized to histone H4 and input DNA, respectively.

#### SAHF composite profiles

To generate SAHF enrichment profiles, the area between each intersected late replicating region and H3K9me3 peak was divided as the H3K9me3 peak enrichment composite profiles. The mean difference (replicative senescent minus proliferating or OIS minus control) in normalized ChIP-seq signal and percentage methylated CpGs was then calculated for each window. H4K20me3, H4K16ac, and H3K9me3 were normalized to histone H4, histone H4, and input DNA, respectively.

#### Spearman correlation coefficients for expression versus ChIP enrichment

To generate Spearman values the ChIP-seq signal was calculated at the gene body of all genes within the gene set. Spearman values were calculated using the R (v3.0.2) method cor.test.

#### Overlap plots

The overlap between two sets of regions was determined using the bedtools tool intersectBed under default parameters [88].

#### Observed-to-expected overlap

Overlaps were computed on a per base pair basis between two datasets (A and B). For every region within A, the number of base pairs that were occupied by a region within B was computed. A permutation test was performed in order to determine the background genomic average expected overlap. We generated 10,000 sets of regions with properties (length distribution and chromosome distribution) equal to set B. Randomly generated regions of B were prevented from being generated within unsequenced regions of the genome (as defined by the UCSC mapping and sequencing track - "gap"). The overlap of A and B was repeated for each randomly generated set of B to determine the average expected random overlap. *P* values were estimated empirically from the observed overlaps of the randomly generated sets.

#### Genomic features

Coding genes were defined as all Ensembl genes (version 73) of Gene Biotype protein coding and status known. Promoters were defined as the region spanning ±2 kb of the outermost transcription start site of each coding gene. Exons, 3′ UTRs, and 5′ UTRs were defined as the corresponding Ensembl (version 73) regions for known coding genes, and introns as the corresponding genic but not intronic regions. RNA genes were defined as all Ensembl non-coding, non-pseudo genes. Repetitive elements and CpG islands were obtained from UCSC (hg19). CpG island shores were defined as the 2 kb flanking the CpG island and CpG island shelves as the 2 kb flanking the shores [90]. Hypermethylated and hypomethylated regions were obtained from GEO (http://www.ncbi.nlm.nih.gov/geo/query/acc.cgi?acc=GSE48580 = GSE48580) and converted to hg19 using UCSCs liftover tool [89]. Early/late replicating regions were mapped previously [21] and converted to hg19 using UCSCs liftover tool [89].

#### H4K20me3 abundance at telomeres by ChIP-seq

To map telomeric reads, a 1-kb telomeric repeat (TTAGGG) sequence was generated and reads aligned using the Bowtie2 alignment software. The percentage of mapped reads for H4K20me3, histone H4, and input was calculated for proliferating and senescent cells. The ratio of percentage mapped reads H4K20me3/percentage mapped reads control was then plotted.

#### H4K20me3 enrichment at TE subtypes by evolutionary order

To determine the enrichment at TE subtypes, for each set of DiffBind peaks, the observed/expected fold overlap with each TE subtype was calculated. TE subtypes were obtained from UCSC. Next, the evolutionary order of each TE subtype was determined as described previously [62].

#### The Cancer Genome Atlas SUV420H2 expression

The Cancer Genome Atlas (TCGA; http://cancergenome.nih.gov) SUV420H2 RNA-seq expression Z scores for each cancer type and sample was obtained from cBIOPortal (http://www.cbioportal.org/about_us.jsp). Boxplots in Fig. 7c were generated using R (v3.0.2). The bottom and top of the boxes correspond to the 25th and 75th percentiles respectively, and the internal band is the median. The plot whiskers correspond to the most extreme value within 1.5 x interquartile range.

#### cBIOPortal definition of Z scores

For mRNA and microRNA expression data, we typically compute the relative expression of an individual gene and tumor to the gene’s expression distribution in a reference population. The reference population was either all tumors diploid for the gene in question or, when available, normal adjacent tissue. The returned value indicates the number of standard deviations away from the mean of expression in the reference population (Z score). This measure is useful in determining whether a gene is up- or downregulated relative to the normal samples or all other tumor samples.

### Xenograft experiments

Five-week-old, female CD-1 athymic nude mice (Crl:NU-*Foxn1*^*nu*^) were obtained from Charles River Laboratories and equilibrated to the institute animal facility for 1 week. At 6 weeks of age, mice were injected subcutaneously in the flank with 2 × 10^6^ HT1080 cells stably infected with either control or SUV420H2 retrovirus and suspended in 100 μl sterile PBS. Mice were monitored daily for any adverse clinical signs and tumor size measurements obtained approximately every two days using manual calipers. Mice were culled when tumors reached 10 mm in any dimension and tumors were collected for histopathological and biochemical evaluation. To calculate tumor volume (mm^3^), the length (L) and width (W) of the tumor was measured with calipers and tumor volume calculated from L × W × W (where W is the smaller of the two measurements).

## Abbreviations

5-BrdU, 5-bromo-2′-deoxyuridine; ChIP, chromatin immunoprecipitation; ChIP-seq, chromatin immunoprecipitation-sequencing; DMEM, Dulbecco’s modified Eagle’s medium; EdU, 5-ethynyl-2′-deoxyuridine; GEO, Gene Expression Omnibus; IP, immunoprecipitation; IPDB, immunoprecipitation dilution buffer; LAD, lamin associated domain; LINE, long interspersed nuclear element; LTR, long terminal repeat; mNLB, modified nuclear lysis buffer; OIS, oncogene-induced senescence; qPCR, quantitative PCR; RS, replicative senescence; SA β-gal, senescence-associated β-galactosidase; SAHF, senescence-associated heterochromatin foci; SASP, senescence-associated secretory phenotype; TE, transposable element

## Additional files

Additional file 1: Six additional supplementary figures and legends. (PDF 10.1 MB) Additional file 2: Table S1. Descriptive statistics for H4K20me3, histone H4, and input DNA ChIP sequencing reads from proliferating (PRO) and RS, control (CON) and OIS, and CON and H2 IMR90 cells. **Table S2.** Descriptive statistics for RNA sequencing reads from PRO, RS, CON and OIS IMR90 cells. (DOC 75 kb) Additional file 3: Dataset 1. Ranked list of the 500 genes containing the greatest H4K20me3 enrichment in RS cells. **Dataset 2.** Ranked list of the 500 genes containing the greatest H4K20me3 enrichment in OIS cells. (XLS 109 kb)
