# Supplementary material for: Mapping H4K20me3 onto the chromatin landscape of senescent cells indicates a function in control of cell senescence and tumor suppression through preservation of genetic and epigenetic stability
Source: Genome Biol. 2016 Jul 25;17:158. doi: 10.1186/s13059-016-1017-x (PMC4960804; doi:10.1186/s13059-016-1017-x)
Supplement: Additional file 1: — Six additional supplementary figures and legends. (PDF 10.1 MB) [file 13059_2016_1017_MOESM1_ESM.pdf]

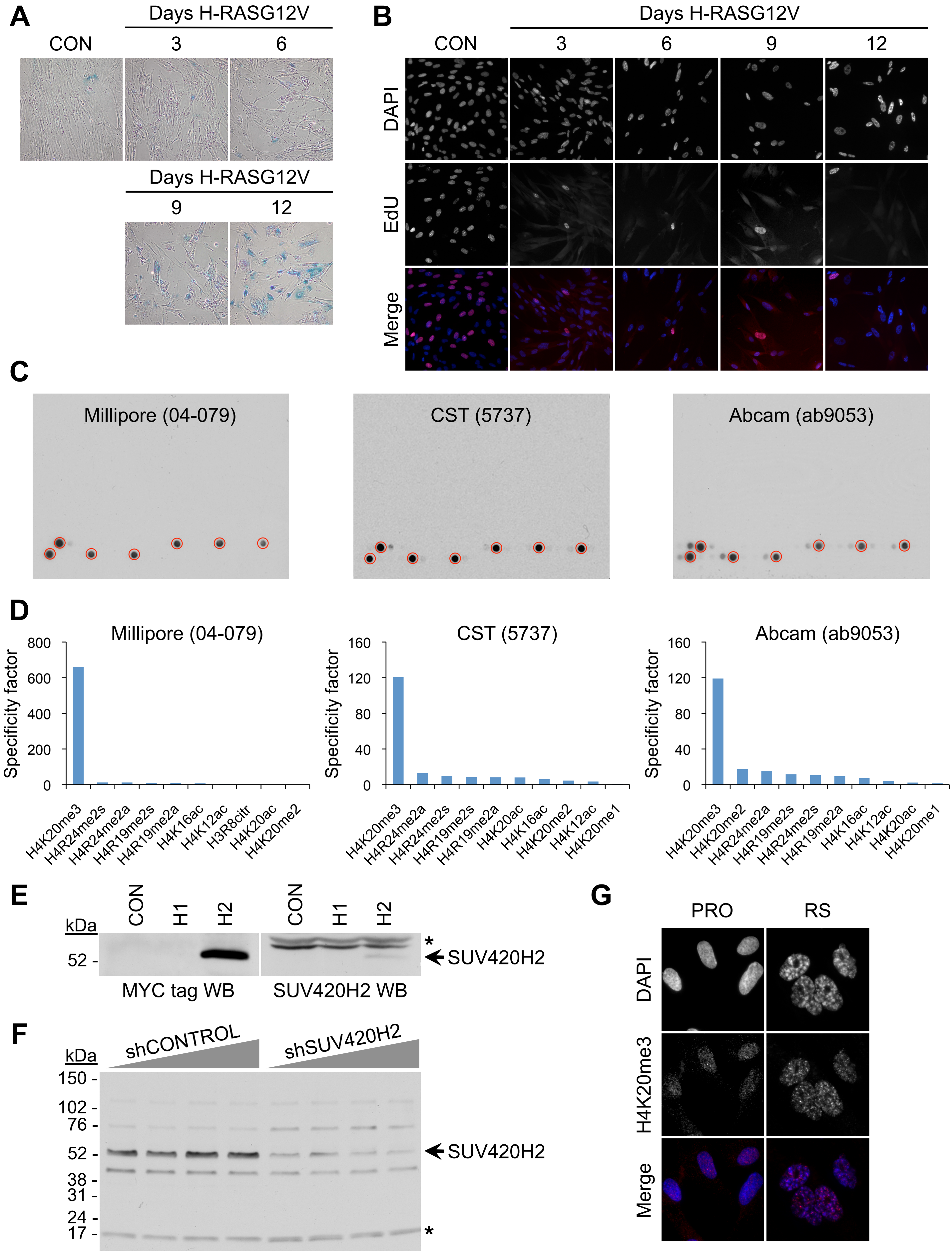

Supplementary Figure S1

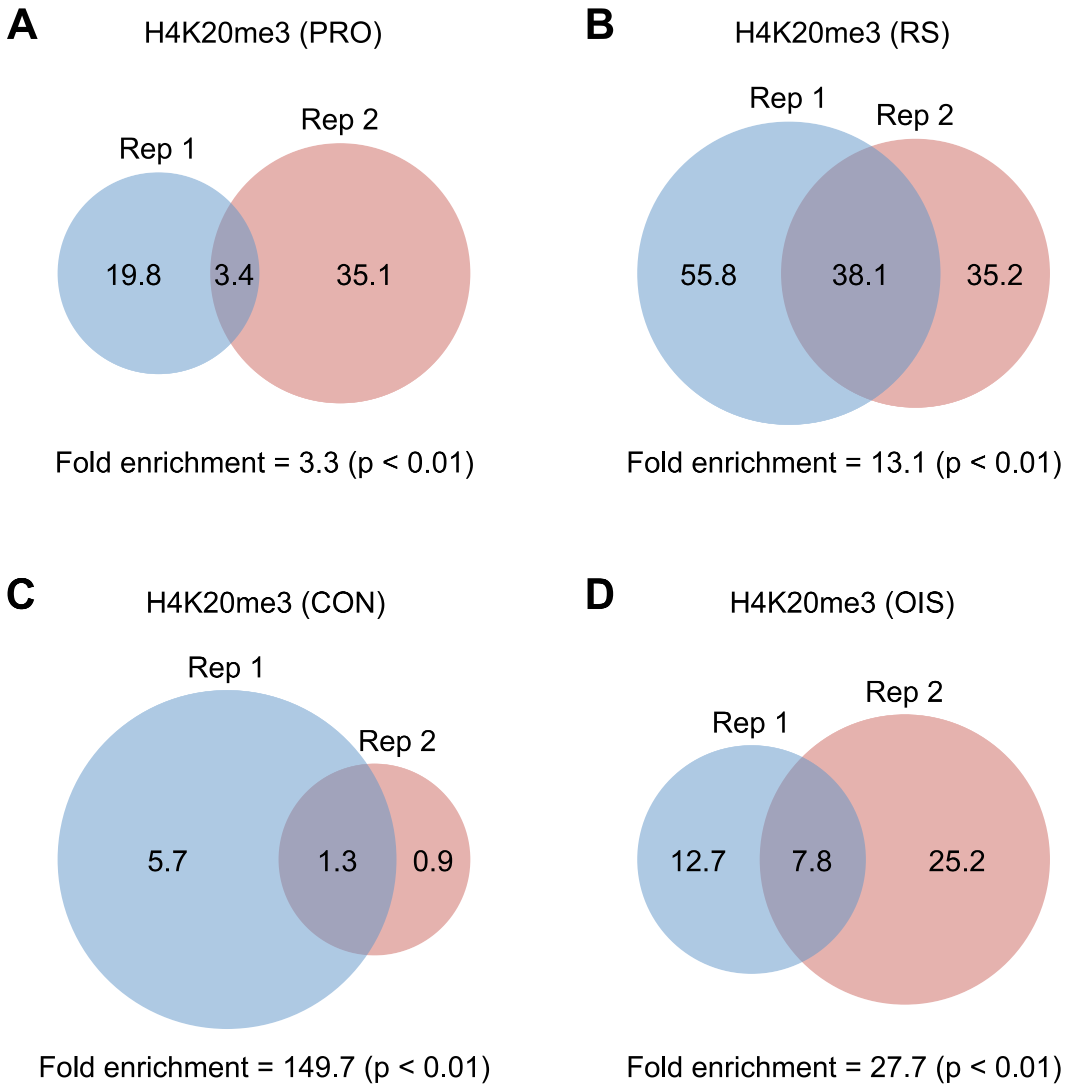

Supplementary Figure S2

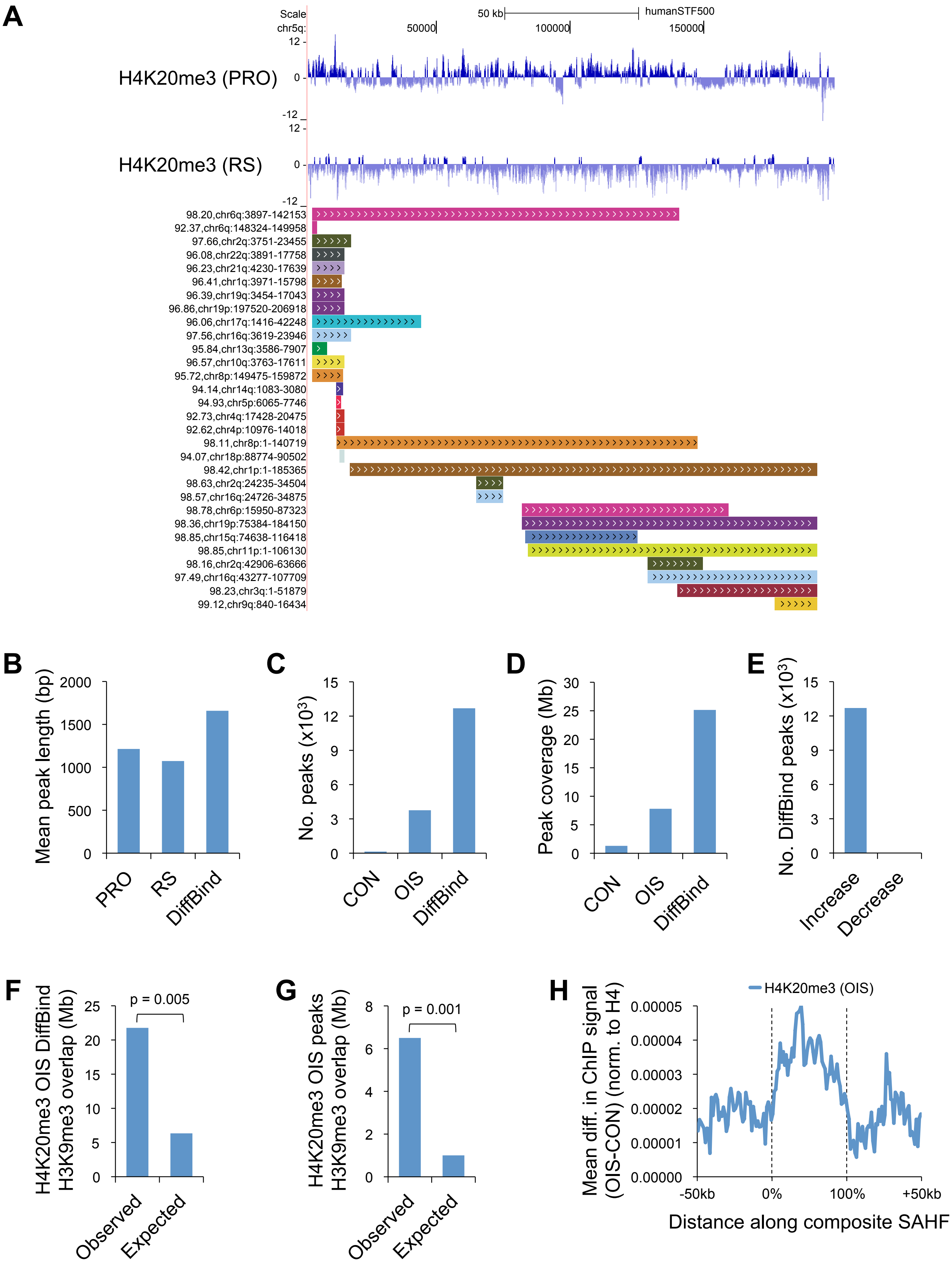

Supplementary Figure S3

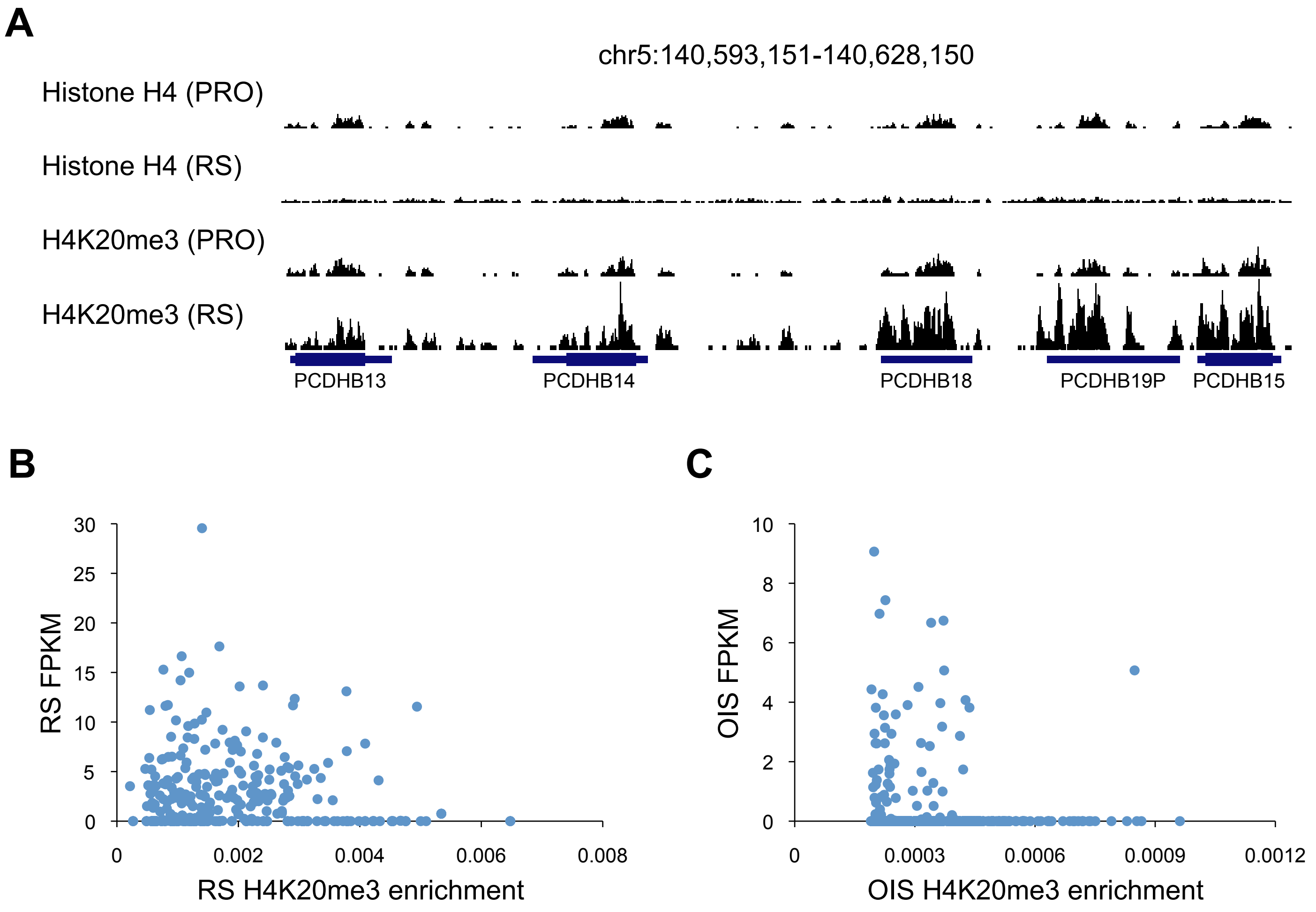

Supplementary Figure S4

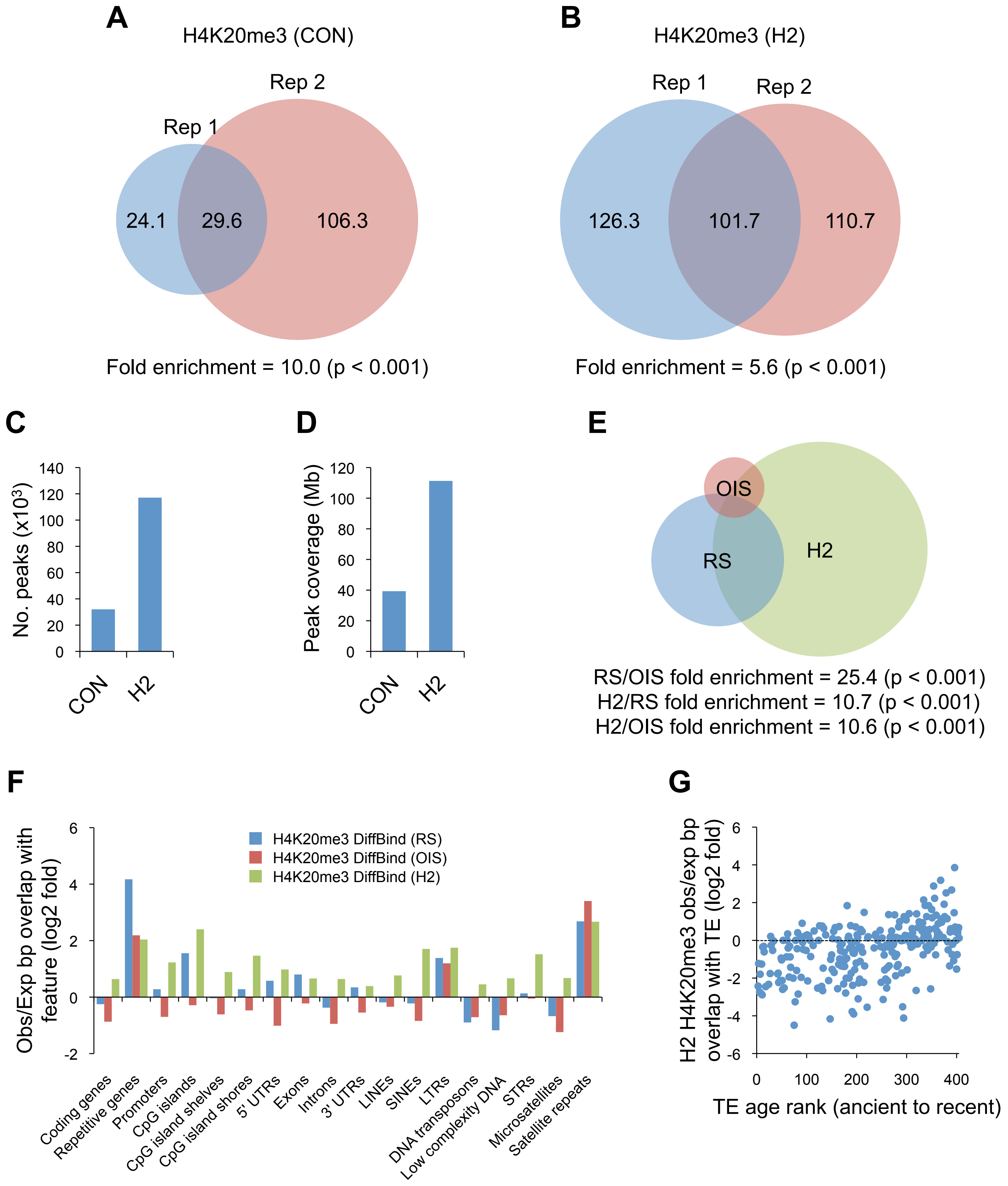

Supplementary Figure S5

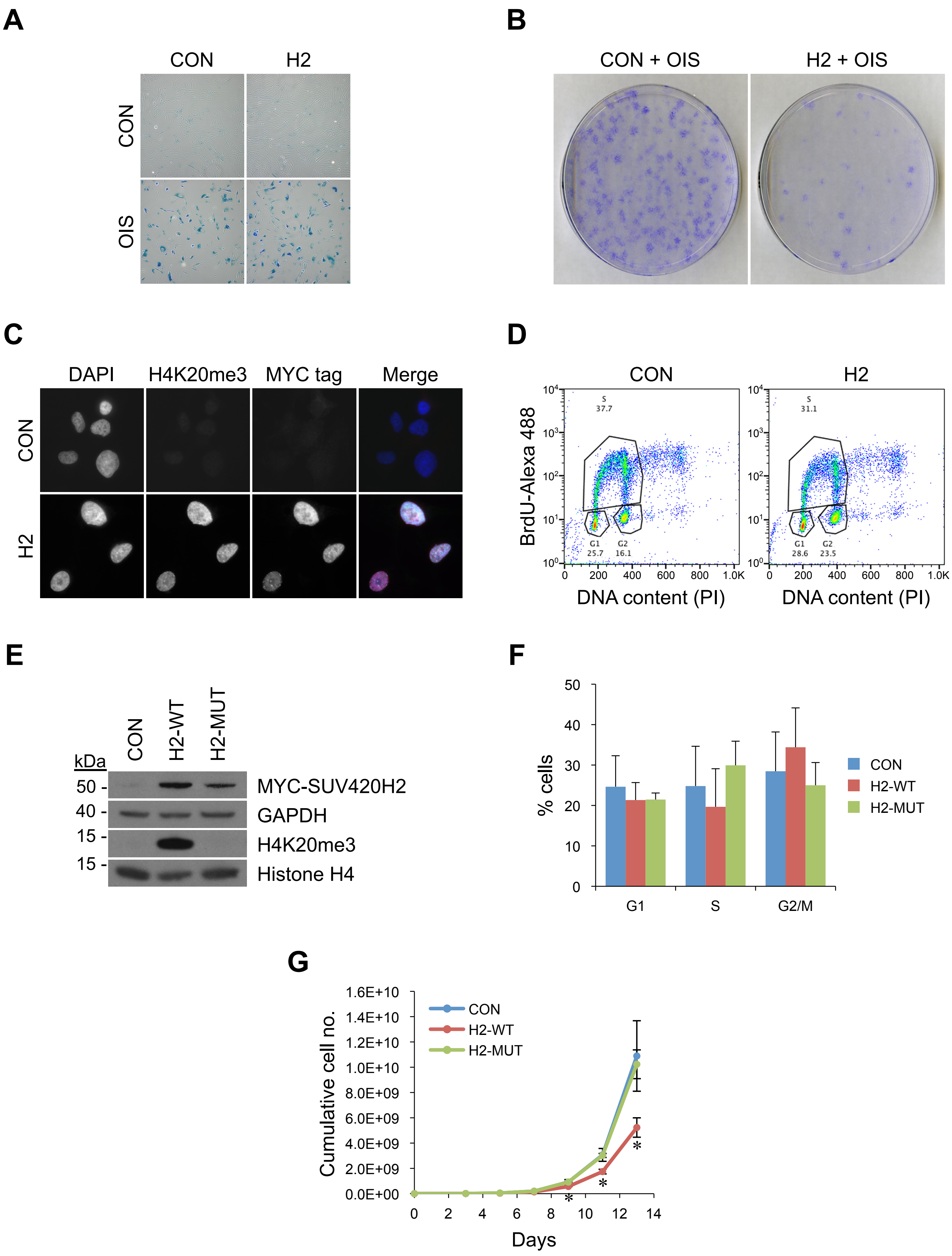

Supplementary Figure S6

Figure S1. Related to Fig. 1. **a** Senescence-associated β-galactosidase staining in IMR90 cells 3–12 days after infection with either empty vector control (*CON*) or H-RASG12V retrovirus. **b** Cells from **a** were pulse labeled with 5-ethynyl-2′-deoxyuridine (EdU) and visualized using Click-iT® detection and fluorescence microscopy. **c** Autoradiographic images of MODified Histone Peptide Array (Active Motif) analysis of three H4K20me3 antibodies (Millipore, 04–079; Cell Signaling Technology, 5737; Abcam, ab9053); all spots on the array containing H4K20me3 peptides are indicated by *red circles*. Most peptide spots on the array (other modifications) are not detected. For the array key, see http://www.activemotif.com/catalog/667/histone-peptide-array. **d** Antibody binding specificity (specificity factor) was calculated for the antibodies from **c** using Array Analyse (Active Motif); for each antibody, peptides were ranked from greatest to least degree of binding. **e** Western blot of ectopically expressed, Myc-tagged SUV420H2 from whole cell extracts of IMR90 cells infected with empty vector control (*CON*), MYC-SUV420H1 (*H1*), or MYC-SUV420H2 (*H2*) virus using antibodies against either the Myc tag or SUV420H2. Non-specific bands (*asterisk*) indicate equal loading. **f** Western blot of SUV420H2 from whole cell extracts of IMR90 cells infected with increasing amounts (indicated by *filled triangles*) of either empty vector control shRNA (*shCONTROL*) or shRNA targeting SUV420H2 (*shSUV420H2*) four days after infection. Non-specific bands (*asterisk*) indicate equal loading. **g** Immunofluorescent images of H4K20me3 staining in proliferating (*PRO*) and RS IMR90 cells.

Figure S2. Related to Fig. 3. **a** Proportional Venn diagram of H4K20me3 peak coverage (Mb) obtained with Millipore 04–079 (*Rep 1*) and Cell Signaling Technology 5737 (*Rep 2*) antibodies in proliferating (*PRO*) IMR90 cells. The fold and significance of overlap compared with random is indicated. **b** As in **a** but with replicative senescent (*RS*) IMR90 cells. **c** Proportional Venn diagram of H4K20me3 peak coverage (Mb) obtained with Millipore 04–079 antibody from two replicate experiments in IMR90 cells infected with empty vector control (*CON*) retrovirus. **d** As in **c** but with H-RASG12V retrovirus-infected senescent (*OIS*) IMR90 cells.

Figure S3. Related to Fig. 3. **a** UCSC Genome Browser view of aligned H4K20me3 reads (normalized to total number of reads per lane) at chromosome 5q subtelomeric repeat elements in proliferating (*PRO*) and RS cells. 0 bp (left of *x-axis*) is the transition between subtelomeric and telomeric repeats (not shown). Specific subtelomeric repeat elements are indicated by *colored horizontal bars* (*below*). The data are graphed as log fold change in the mapped H4K20me3 reads relative to the mapped H4 reads in the control dataset after correcting for the (slightly) different number of total reads in the two datasets. The vertical scale was set at 12 for this viewing window to allow direct comparison of the relative enrichments between the datasets from PRO and RS cells. We used multi-read mapping parameters as described in [1], where uniquely mapping reads are given a value of 1 and multiply mapping reads are given a fractional value dependent upon how many sites the read maps to in the genome (e.g., 0.25 if it maps to four sites, 0.125 if it maps to eight sites, etc.). The multiread mapping parameters are essential for mapping short-read datasets in low-copy segmental duplication regions such

as subtelomeric repeat regions [1]. **b** Mean peak length (bp) of H4K20me3 peaks identified with both antibodies (intersection) in PRO and RS cells and significantly different (FDR < 0.01) peaks between PRO and RS, determined by DiffBind. **c** Total number of overlapping H4K20me3 peaks identified in both replicates (intersection) in control (*CON*) and OIS cells and significantly different (FDR < 0.01) peaks between CON and OIS, determined by DiffBind. **d** Total number of base pairs comprising H4K20me3 peaks identified in both replicates (intersection) in CON and OIS cells and significantly different (FDR < 0.01) peaks between CON and OIS, determined by DiffBind. **e** Number of H4K20me3 DiffBind peaks from **c** that increase and decrease in OIS cells relative to CON cells. **f** Observed overlap and expected overlap (enrichment compared with random) between base pairs covered by OIS H4K20me3 DiffBind peaks with base pairs covered by H3K9me3 peaks in senescent cells (empirical $p < 0.001$). **g** Observed overlap and expected (enrichment compared with random) overlap between base pairs covered by OIS H4K20me3 peaks (intersection of both replicates) with base pairs covered by H3K9me3 in senescent cells (empirical $p < 0.001$). **h** Mean difference (OIS − CON) in H4K20me3 enrichment at a composite H3K9me3-marked late replicating region.

Figure S4. Related to Fig. 4. **a** UCSC Genome Browser view of histone H4 and H4K20me3 ChIP-seq reads from proliferating (*PRO*) and RS cells aligned along a 35-kb segment of chromosome 5 containing a protocadherin gene cluster. Duplicate aligned reads have been removed but reads have not been filtered for unique alignment. Data are normalized to library size. **b** Scatter plot comparing RS H4K20me3 enrichment versus RS expression (FPKM) of the portion of the top 500 H4K20me3 enriched genes that are also in repeat gene classes (309/500 genes). **c** Scatter plot comparing OIS H4K20me3 enrichment versus OIS expression (FPKM) of the portion of the top 500 H4K20me3 enriched genes that are also in repeat gene classes (305/500 genes).

Figure S5. Related to Fig. 7. **a** Proportional Venn diagram of H4K20me3 peak coverage (Mb) obtained with Millipore 04–079 (*Rep 1*) and Cell Signaling Technology 5737 (*Rep 2*) antibodies in IMR90 cells infected with empty vector control retrovirus (*CON*). **b** As in **a** but in IMR90 cells infected with MYC-tagged SUV420H2 retrovirus (*H2*). **c** Total number of overlapping H4K20me3 peaks identified in both replicates (intersection) in CON and H2 cells. Peaks were normalized against input DNA. **d** Total number of base pairs comprising H4K20me3 peaks identified in CON and H2 cells. **e** The total base pair overlap of the RS, OIS, and H2 H4K20me3 peaks; the significance and fold change over random is indicated. **f** Observed overlap/expected overlap (fold log2) (enrichment compared with random) between base pairs in RS, OIS, and H2 H4K20me3 DiffBind peaks and base pairs covered by specified fixed genomic features. RS and OIS values from Fig. 4a are also shown here for comparison with H2 cells. **g** Observed overlap/expected overlap (fold log2; enrichment compared with random) between base pairs in H2 H4K20me3 DiffBind peaks and TEs. The *x-axis* shows TE evolutionary order ranked from most ancient to most recent.

Figure S6. Related to Figs. 6 and 7. **a** Senescence-associated β-galactosidase staining in control and SUV420H2-expressing IMR90 cells, 15 days after infection with either empty vector control (*CON*) or HRASG12V (*OIS*) retrovirus. **b** Plates of cells from Fig. 6f were stained with crystal violet 30 days after infection. **c** Immunofluorescent images of HT1080 cells infected with vector control (*CON*) or Myc-tagged SUV420H2 (*H2*) retroviruses. **d** Representative gating used for FACS analysis of CON and H2-infected HT1080 cells in Fig. 7e, f. **e** Western blot of indicated proteins from whole cell extracts of HT1080 cells infected with vector control (*CON*), MYC-tagged wild-type SUV420H2 (*H2-WT*) or MYC-tagged catalytically inactive mutant SUV420H2^{N182A,Y217A} (*H2-MUT*) retroviruses. **f** FACS analysis of CON, H2-WT, and H2-MUT HT1080 cells pulse labeled with 5-BrdU, fixed, and stained with propidium iodide to determine proportion of cells in G1, S, and G2/M phases; *error bars* represent standard deviation (n = 3). **g** Growth curves expressed as cumulative cell number for CON, H2-WT, and H2-MUT HT1080 cells measured for 13 days after infection; *asterisks* denote statistically significant difference between H2-WT and H2-MUT ($p < 0.05$).

References.

1. Stong N, Deng Z, Gupta R, Hu S, Paul S, Weiner AK, et al. Subtelomeric CTCF and cohesin binding site organization using improved subtelomere assemblies and a novel annotation pipeline. Genome Res. 2014;24:1039–50.
