# Supplementary material for: Mapping H4K20me3 onto the chromatin landscape of senescent cells indicates a function in control of cell senescence and tumor suppression through preservation of genetic and epigenetic stability
Source: Genome Biol. 2016 Jul 25;17:158. doi: 10.1186/s13059-016-1017-x (PMC4960804; doi:10.1186/s13059-016-1017-x)
Supplement: Additional file 2: Table S1. — Descriptive statistics for H4K20me3, histone H4, and input DNA ChIP sequencing reads from proliferating (PRO) and RS, control (CON) and OIS, and CON and H2 IMR90 cells. Table S2. Descriptive statistics for RNA sequencing reads from PRO, RS, CON and OIS IMR90 cells. (DOC 75 kb) [file 13059_2016_1017_MOESM2_ESM.doc]

**Additional file 1**

**Table S1. ChIP sequencing**

| Replicate | Sample | Raw sequence reads | Read length | Aligned reads (% of raw sequence reads) | Uniquely aligned reads (% of aligned reads) | Duplicate reads (% of uniquely aligned reads) |
| --- | --- | --- | --- | --- | --- | --- |
| Replicate 1 | H4K20me3 PRO | 39,484,830 | 75SE | 20,454,475 (51.80%) | 13,573,412 (66.36%) | 1,557,381 (11.47%) |
| Replicate 2 | H4K20me3 PRO | 32,579,665 | 76SE | 31,513,652 (96.73%) | 20,458,553 (64.92%) | 14,041,295 (68.63%) |
| Replicate 1 | H4K20me3 RS | 40,153,145 | 75SE | 11,877,393 (29.58%) | 7,204,019 (60.65%) | 480,160 (6.67%) |
| Replicate 2 | H4K20me3 RS | 35,612,628 | 76SE | 34,004,626 (95.48%) | 21,420,438 (62.99%) | 11,302,493 (52.76%) |
| Replicate 1 | Histone H4 PRO | 38,207,253 | 75SE | 37,518,075 (98.20%) | 23,896,617 (63.69%) | 3,555,752 (14.88%) |
| Replicate 2 | Histone H4 PRO | 173,573,972 | 75SE | 161,745,109 (93.19%) | 108,758,224 (67.24%) | 9,284,667 (8.54%) |
| Replicate 3 | Histone H4 PRO | 36,143,799 | 76SE | 35,122,945 (97.18%) | 22,318,823 (63.54%) | 3,114,353 (13.95%) |
| Replicate 1 | Histone H4 RS | 41,815,564 | 75SE | 40,754,439 (97.46%) | 29,783,168 (73.08%) | 1,631,878 (5.48%) |
| Replicate 2 | Histone H4 RS | 95,517,411 | 75SE | 93,106,290 (97.48%) | 62,064,893 (66.66%) | 4,626,073 (7.45%) |
| Replicate 3 | Histone H4 RS | 39,250,360 | 76SE | 37,943,697 (96.67%) | 27,509,615 (72.50%) | 1,422,789 (5.17%) |
| Replicate 1 | H4K20me3 CON | 52,402,142 | 72SE | 49,391,035 (94.25%) | 46,357,610 (93.86%) | 3,033,425 (6.54%) |
| Replicate 2 | H4K20me3 CON | 46,950,322 | 72SE | 38,947,474 (82.95%) | 35,986,674 (92.40%) | 2,960,800 (8.23%) |
| Replicate 1 | H4K20me3 OIS | 54,300,630 | 72SE | 28,708,509 (52.87%) | 25,801,955 (89.88%) | 2,906,554 (11.26%) |
| Replicate 2 | H4K20me3 OIS | 39,863,734 | 72SE | 20,398,500 (51.17%) | 18,182,817 (89.14%) | 2,215,683 (12.19%) |
| Replicate 1 | Histone H4 CON | 62,804,180 | 72SE | 61,207,528 (97.46%) | 57,460,088 (93.88%) | 3,747,440 (6.52%) |
| Replicate 2 | Histone H4 CON | 64,589,146 | 72SE | 62,812,618 (97.25%) | 57,641,150 (91.77%) | 5,171,468 (8.97%) |
| Replicate 1 | Histone H4 OIS | 46,680,952 | 72SE | 45,089,853 (96.59%) | 41,551,406 (92.15%) | 3,538,447 (8.52%) |
| Replicate 2 | Histone H4 OIS | 51,330,312 | 72SE | 49,797,923 (97.01%) | 44,104,533 (88.57%) | 5,693,390 (12.91%) |
| Replicate 1 | H4K20me3 CON | 37,776,092 | 72SE | 19,711,923 (52.18%) | 13,995,410 (71.00%) | 5,716,513 (40.85%) |
| Replicate 2 | H4K20me3 CON | 34,551,204 | 72SE | 32,675,481  (94.57%) | 19,184,031 (58.71%) | 13,491,450 (70.33%) |
| Replicate 1 | Input  CON | 45,405,621 | 72SE | 44,016,168 (96.94%) | 42,163,836 (95.79%) | 1,852,332 (4.39%) |
| Replicate 1 | H4K20me3 H2 | 39,074,862 | 72SE | 30,571,784 (78.24%) | 24,040,372 (78.64%) | 6,531,412 (27.17%) |
| Replicate 2 | H4K20me3 H2 | 38,978,674 | 72SE | 36,232,057 (92.95%) | 29,406,635 (81.16%) | 6,825,422 (23.21%) |
| Replicate 1 | Input  H2 | 40,825,214 | 72SE | 39,467,127 (96.67%) | 37,678,964 (95.47%) | 1,788,163 (4.75%) |

**Table S2. RNA sequencing**

| Replicate | Sample | Raw sequence reads | Read length | Aligned reads (% of raw sequence reads) | Duplicate reads (% of aligned reads) |
| --- | --- | --- | --- | --- | --- |
| Replicate 1 | PRO | 46,751,313 | 72PE | 39,062,466 (83.55%) | 13,100,746 (33.54%) |
| Replicate 2 | PRO | 49,874,009 | 72PE | 41,996,835 (84.21%) | 13,935,254 (33.18%) |
| Replicate 1 | RS | 39,939,645 | 72PE | 33,171,173 (83.05%) | 11,747,697 (35.42%) |
| Replicate 2 | RS | 49,374,817 | 72PE | 42,527,566 (86.13%) | 13,852,934 (32.57%) |
| Replicate 1 | CON | 71,663,726 | 72PE | 49,709,871 (69.37%) | 15,947,715 (32.08%) |
| Replicate 2 | CON | 87,704,544 | 72PE | 60,821,604 (69.35%) | 19,646,345 (32.30%) |
| Replicate 1 | OIS | 65,724,432 | 72PE | 42,641,297 (64.88%) | 16,417,415 (38.50%) |
| Replicate 2 | OIS | 83,580,956 | 72PE | 54,045,859 (64.66%) | 21,269,855 (39.36%) |
